# Recent progress in cryoablation cancer therapy and nanoparticles mediated cryoablation

**DOI:** 10.7150/thno.67530

**Published:** 2022-02-14

**Authors:** Kijung Kwak, Bo Yu, Robert J. Lewandowski, Dong-Hyun Kim

**Affiliations:** 1 Department of Radiology, Feinberg School of Medicine, Northwestern University, Chicago, IL 60611, USA.; 2 Department of Biomedical Engineering, McCormick School of Engineering, Evanston, IL 60208, USA.; 3 Robert H. Lurie Comprehensive Cancer Center, Chicago, IL 60611, USA.; 4 Department of Bioengineering, University of Illinois at Chicago, Chicago, IL 60607, USA.

**Keywords:** cryoablation, nanoparticles, image-guided therapy, ablation, immunotherapy

## Abstract

With rapid advances in modern imaging, minimally invasive ablative procedures have emerged as popular alternatives to surgical removal of tumors. Tumor ablation modalities currently offered in clinical practice include microwave ablation, radiofrequency ablation, cryoablation, high-intensity focused ultrasound, and irreversible electroporation. Cryoablation, a non-heat-based method of ablation, is increasingly being used for treating various solid tumors. Accumulated comparative data of cryoablation versus heat-based ablation techniques (e.g., radiofrequency and microwave ablation) shows superior tumor response and quicker recovery time. Evolving research has demonstrated that nanocarriers may serves as excellent catalysts for the cryoablation therapy, imaging guidance, and the co-delivery of therapeutics for minimally invasive, precise, and complete treatment of cancer with immune modulation. This review article focuses on the current status of cryoablation in clinical practice, considers opportunities for enhancing therapeutic outcomes from cryoablation, and discusses new research in the field, including theranostic nanoparticles-mediated cryotherapy and combinational cryo-based immunotherapies.

## Introduction

Since the rapid advancement of ablation-based technology in the 1990s, energy-based ablation has been used for the treatment of many tumor types, including liver, kidney, lung, breast, and prostate cancers, and soft tissue sarcomas. Being minimally invasive, they are now primarily used for the treatment of small, unresectable tumors or for patients who are poor surgical candidates. Cryoablation has distinct advantages over other modes of ablation (radiofrequency, microwave, ultrasound, irreversible electroporation) in that it can be monitored with imaging during the procedure, have greater control of the shape of the ablation zone, and mitigate pain. Among cancer therapies, cryoablation is well-positioned for precision image-guided surgery, which has shown to reduce complications, costs, and recovery time 1, 2. It is also considered a prime candidate to achieve synergy with adjuvant therapies (e.g., chemotherapy, immunotherapy, radiotherapy, etc.) for complete local destruction of cancer tissue. Various cryoablation-based combination therapies with cutting-edge medical imaging have been tested extensively to advance current ablation regimen in preclinical and clinical trials. Recent reports of cryoablation-mediated systemic anti-tumor immune response introduce new opportunities for immuno-cryotherapy 3. Importantly, recent development of multifunctional nanocarriers can play a key role for the expansion of cryoablation in the treatment of cancer. It is possible to achieve enhanced cryo-cell death and customization of ice formation to fit irregular tumor shapes with noble nanomaterials. Preferably, the course of treatment for local cancer therapy entails image-guided delineation of tumor regions and seeding nanoparticles in various locations to increase (in the tumor) or reduce (around healthy tissues) ice formation. Combination adjuvant therapy and cryoablation can be directed with imaging to visualize ablation zones during and after the operation. With these advantages, nanoparticle-based combinational cryotherapy can overcome barriers present in traditional chemotherapy or cryoablation monotherapy, such as drug resistance (chemotherapy), systemic toxicity, and heterogenous destruction of cancer cells. This review will provide the current status, progress, and promise of cryoablation and cryoablation-based combination therapies, including the future application of multifunctional nanocarriers with cryoablation.

## Cryoablation Cancer Therapy and Action Mechanism

Although the application of cold temperatures to treat fractures and wounds have been traced back to 3000 B.C., the first application of cryotherapy in tumor was carried out by Dr. James Arnott in the mid-19^th^ century 4. Currently, the most common cooling method of cryoablation involves circulating cooled fluids such as nitrogen or argon through probes 3, which then rapidly expand into gas, creating temperatures as low as -190 °C in what is known as Joule-Thomson effect 5. While argon and helium have been widely used in cooling the cryo-probe, the accessibility and cost of the noble gases have delayed the application of cryoablation treatment in low and middle-income countries 6. To circumvent this problem, a low-cost carbon dioxide-based cryoablation device was developed 7. Highly pressurized carbon dioxide gas can expand and cool to temperatures as low as -78.5 °C, creating an environment sufficient for ice formation and cellular necrosis. In rats with hepatic tumors, complete cryoablation is achieved at temperatures -38 °C or lower at the edge of the ice balls formed 8.

**Figure 1:**
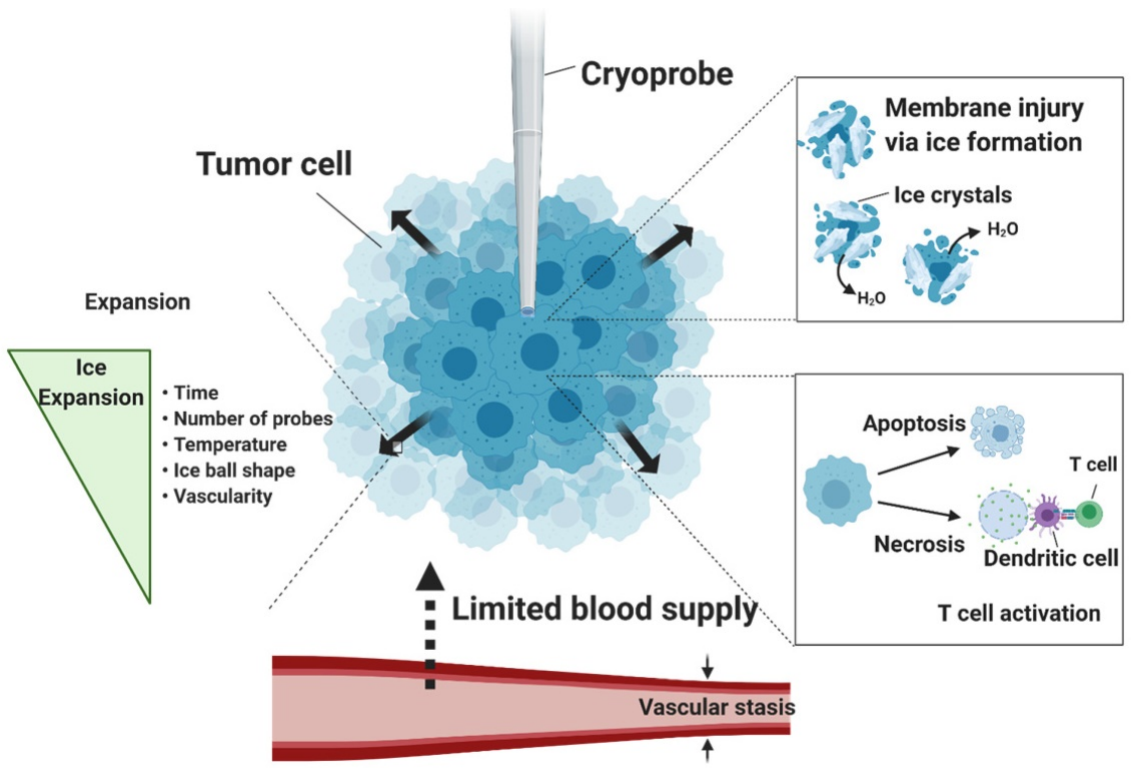
** Mechanisms of cryoablation.** At extreme temperatures below -20 °C, intracellular ice forms and the subsequent cell death occurs in two forms: necrosis and apoptosis. Necrosis results from damaged cellular membranes, releasing tumor antigens that are phagocytosed by dendritic cells. In apoptosis, disturbance of mitochondrial activities causes initiation of Bax proteins, which triggers downstream apoptotic pathways. Besides the two principal mechanisms of cell death, the formation of ice ball depends on many factors, including the duration of freeze-thaw cycle, number of cryoprobes, temperature, ice ball shape, and vascularity of the target ablation zone.

Cellular death by cryoablation is accomplished through multiple mechanisms, including physical damage of cellular membrane by ice formation, initiation of cellular stress responses (kill switch activation) and necrosis and apoptosis cascade, vascular stasis during thawing process, and likely activation of immune responses (**Figure 1**) 9. The main mechanism of ablation occurs through the formation of intracellular ice crystals below temperatures -20 °C. Ice crystal formation incurs mechanical stress on membranes and freezing-thawing cycles disturb metabolic functions of the mitochondria. Destruction of the membranes results in necrosis and the release of cellular content, including damage-associated molecular patterns (DAMPs) and tumor-specific antigens which are taken up by peripheral macrophages for further immune activation. On the other hand, disturbance of mitochondrial functions triggers apoptosis. A rise in pro-apoptotic Bax proteins immediately after thawing signals initiation of apoptotic pathway in the affected cells. Further, loss of blood supply to the tumor from vascular stasis increases damage to the cells.

Several factors besides low temperature promote complete ablation, such as the number and position of the cryoprobes, the shape of ice ball optimal for covering the entire tumor 10, depth of ice penetration, number of freeze-thaw cycles, and freezing time. According to the thermodynamic properties of cryoablation modelled in atrial fibrillation, the ice front penetration is proportional to the square root of time while its velocity depends on the heat subtracted flux during the ice formation, and prolonged freezing time kills surrounding tissues 11. Although there is no agreed standard duration of cryoablation among physicians, typically longer freeze and thaw durations result in greater cell death. In a preclinical canine study treating atrial fibrillation, cryoablation was found to be most effective within 30 to 60 seconds 12. In humans, single 3-minute freeze cryoablation was successful in 42.9% of the patients without major complications at the mid-term follow-up of 26.5 ± 13.7 months 13. Along with freezing duration, the number of freeze-thaw cycles also affects the cytotoxic potential of the cryoablation treatment. Triple-freeze (5-5-5-5-10-5) versus dual-freeze (10-5-10-5) cryoablation was evaluated in a pig model 14. Within the same freezing time of 20 minutes, the triple-freeze protocol produced a larger zone of complete necrosis than the dual-freeze protocol, although the length of the zone and cryolesions were similar.

Vascularity also affects the area of cryolesions. Like the heat sink effect, warm blood flows into locally cooled tissues and raises temperature during cryoablation so it becomes difficult to cover the entire region of diseased tissues with the ice ball. Computational studies of the effects of arterial bifurcation (branching) on the temperature distribution during cryosurgery have shown that the convective heat transfer from bifurcated vessels can limit ice ball formation, cause irregular ice ball shape near the arterial bifurcation and prevent the blood vessels from being frozen 15. Hence, areas of tissue that have high vascularity may see less benefit from cryoablation, although artery walls may benefit from lower susceptibility to ice-induced damage. To mitigate the heat transfer, embolization of blood vessels to prevent blood flow may increase the efficacy of cryoablation while delivering ischemic cytotoxicity to tumor cells. In a Danish Landrace pig model, arterial clamping to artificially reduce renal blood flow increased cryolesion size by approximately 80%, while seeing no sign of injury in the kidney tissues due to limited blood flow model 16.

## Cryoablation for the treatment of various types of cancers in the clinic

Cryoablation is currently used to treat benign and malignant tumors of the liver, prostate, kidney, lung, breast, and bone, as well as non-tumor diseases relating to the heart. Advantages and disadvantages of cryoablation in three major organs, liver, prostate, and kidneys, are included in **Table 1** and discussed below in detail.

### Liver Cancer

In certain carcinomas such as liver 39, 40, lung 41-43, renal 44, prostate 45, 46, breast 47, and gastrointestinal 48 cancer where only a low percentage (5-15%) of tumors are amenable to surgical removal, or cryoablation is a potentially curative treatment option for patients with small tumors 49-51. Cha et al. showed that cryoablation was effective in patients with hepatocellular carcinoma (HCC) and metastatic colorectal adenocarcinoma who were not amenable to resection: the overall survival (OS) between the cryoablation group and the cryoablation group combined with resection did not significantly differ, with lower complication rate occurring in the cryoablation group than the latter group 18. Similar success was seen in treating liver metastases from ovarian cancer 52. The size of the liver tumor is important; compared to tumors smaller than 4 cm, cryoablation of hepatic tumors exceeding 4 cm portends decreased technical efficacy rate (60% vs 93.4%), higher rate of local tumor progression (63.3% vs 23.3%), and more Grade 3 or greater adverse events 26. In 98 patients treated with hepatic cryoablation, 11% had major complications and the rate increased significantly with larger treated areas (>30 cm^2^; p = 0.003) 53. Further, large-scale hepatic cryoablation of 30% to 35% of liver parenchyma in sheep and transgenic mice models have resulted in systemic complications, including acute lung injury, increased pulmonary artery pressure and pulmonary capillary permeability 20-25 and has been linked with increased thromboxane levels and NF-κB-mediated inflammatory mechanisms in the lung, liver, and kidney. Despite complication risks in larger tumors, cryoablation can be done safely in a localized fashion on smaller hepatic tumors located close to other organs such as the gallbladder: no gallbladder complications were seen following cryoablation of hepatic tumors (mean size 2.7 cm) within 1 cm of the gallbladder, with the ice ball extending into the gallbladder lumen (95% of cases). In this study, the overall complication rate was 29% with technical success rate of 90%, with 87% of patients having no imaging evidence of local tumor progression at 6-month follow-up 19. In addition to favorable clinical outcomes and high technical success, cryoablation provides numbing effect that help patients cope with the procedure. Cryoablation of painful hepatic tumors or pleural and chest wall metastases has been shown to alleviate pain for 5 to 8 weeks after the procedure 54, 55.

### Renal Cell Carcinoma

Renal cryoablation is a minimally invasive alternative to surgery for poor operative candidates or those with multiple renal tumors, with comparable efficacy to nephrectomy 31. A study of 118 patients who underwent percutaneous cryoablation or partial nephrectomy for a single primary renal tumor in a solitary kidney found no significant differences between the two procedures in the rate of complications, renal functional viability, local tumor recurrence, and cancer-specific mortality 56. Similar findings support the OS rate, safety and effectiveness of cryoablation in small-sized renal cell carcinoma (less than 4 cm in diameter) 32-35, 57, 58. In these experiments, complications resulting from surrounding structures were evaluated along with the completeness of eradication of tumor cells. In general, cryoablation of T1 renal masses had lower rate of overall and postoperative complications and superior renal functional preservation compared to partial nephrectomy 36. Oncological outcomes from cryoablation are dependent on tumor size. A retrospective study done on patients with larger T_1b_ renal cell carcinomas showed a 2.5-fold increase in 5-year cancer specific mortality (but similar other cause mortality) when cryoablation was performed compared to partial nephrectomy 38. T_1b_ renal cell carcinoma patients who underwent partial nephrectomy also had a lower rate of local cancer recurrence than patients with cryoablation (laparoscopic or percutaneous) 59. Further, cryoablation of T_1b_ renal cell carcinomas caused frequent complications (grade II or worse) 37, although fewer complications were seen in completely endophytic masses 60. To reduce the rate of complications, several techniques such as hydro-, carbo-, and pneumo-dissection 61, balloon dissection, and probe traction 62 have been proposed to minimize the impact of cryoablation on adjacent non-renal structures.

### Prostate Cancer

Cryoablation of the prostate, although still investigational, has recently emerged as a focal tool for the treatment of localized prostate cancer. Compared with conventional surgical removal, cryoablation allows for decreased hospitalization time, reduced postoperative morbidity, decreased interval for return to daily activities, and reduced overall treatment cost 28. Patients who underwent cryoablation of anterior prostate cancer at 15-month follow-up had no change in sexual function and tested negative for post-operative cancer biopsy 27. Cryoablation for recurrent prostate tumors has been effective in patients after the first-round radiotherapy (successful in 14 out of 21 patients with intra-prostatic cancer and in 2 out of 7 patients with extra-prostatic cancer) 30 or primary cryoablation 29. Complications tend to slightly increase after the second cryoablation compared to the first cryoablation. MRI-guided cryoablation of prostate tumors after the first-round of radiotherapy is similarly associated with a high technical success rate, preservation of quality of life, and local tumor control 63.

### Skin Cancer

Various skin lesions, including non-melanoma basal and squamous cell carcinomas and Kaposi sarcoma have been routinely treated with cryotherapy. Direct application of liquid nitrogen to the site using cotton tips or spray is used most often to treat superficial masses on the surface of the skin. A large-scale study with 2932 patients afflicted by basal or squamous cell carcinomas less than 2 cm in size saw a 98.6% cure rate over a 30-year period with only 5 cases of recurrence 64. Cryotherapy used to treat Kaposi sarcoma has resulted in complete response in 63% of patients 65. The biggest side effect of cryotherapy to skin is skin burn, which can cause scarring and pigmentation changes. Precise instrumentation can help minimize these deficiencies. For example, a thin cryoneedle in which liquid nitrogen is circulated within the needle showed good cosmetic outcomes in intralesional basal cell carcinoma 66.

### Breast Cancer

Cryoablation has been increasingly used to treat breast cancer over the last two decades, ranging from early to late stage disease. Preference for a good cosmetic outcome has led to breast-conserving, minimally invasive procedures such as radiotherapy and ablation therapies in lieu of surgery. Multiple cases have demonstrated the safety and effectiveness of cryoablation for singular, small-sized tumors 67-70. In cases of multifocal lobular carcinoma, cryoablation is not recommended due to a higher risk of fat necrosis and cryoinjury to healthy tissues from extended freezing time 71. A phase II trial in early stage breast cancer less than 2 cm showed 75.9% overall success rate and 92% success rate in patients without multifocal cancer 69, and a more recent prospective trial conducted on patients with breast tumors less than 1.5 cm showed low recurrence rates and 95% of patients reporting satisfaction with cosmetic results 70. Special care should be taken to prevent burns to the sensitive areas around the nipple. Saline hydro-dissection into subcutaneous tissue may be done to insulate the skin from the ice ball.

## Combination Cryoablation Cancer Therapies

**Figure 2:**
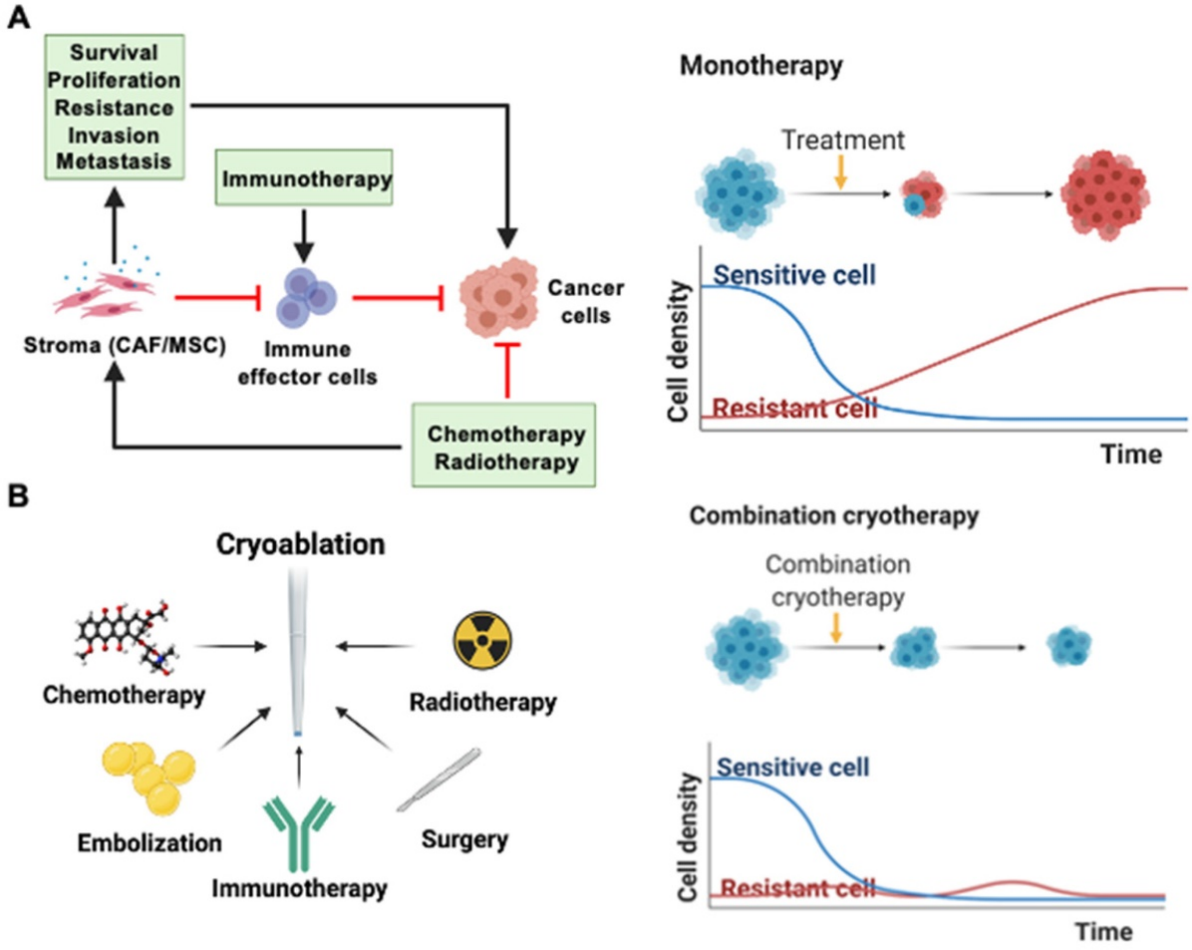
** Synergistic cryo-based combination therapies. (A)** Drug or radiation-induced damage can activate CAFs and MSCs to secrete growth factors that encourage tumor survival, proliferation, resistance, invasion, and metastasis. Immunotherapy can also be hindered by the immunomodulatory effects of the tumor stroma. **(B)** Monotherapies such as chemotherapy, radiotherapy, surgery, embolization, and immunotherapy may be performed jointly with cryoablation. Combination therapies can ensure homogenous destruction of tumor tissues and reduce the likelihood of resistant cancer cells surviving.

Refinements in technology and procedural techniques optimizes the capacity of cryoablation in treating small-sized tumors. However, it is often not sufficient to completely eradicate larger tumors. Combining cryoablation with other cancer therapies may create a synergistic anticancer effect greater than either monotherapy alone. Cryoablation, for instance, can target cancer stem-like cells and create an unfavorable environment for tumor regrowth and vascularization. In the more distal zones of cryoablation, hypoxia and ATP deprivation can promote local acidosis and death of cancer-associated fibroblasts, stifling the exchange of essential growth factors for tumor proliferation and revascularization 72. This can limit the probability of tumor cells surviving to gain resistance to cancer therapies such as chemotherapy or radiotherapy. The tumor stroma, which includes cancer-associated fibroblasts (CAFs), mesenchymal stroma cells (MSCs), and the extracellular matrix (ECM), supports tumor growth through physical and chemical barriers to anti-tumor agents (**Figure 2A**). Drug or radiation-induced DNA damage can induce stromal secretion of factors that promote survival, proliferation, resistance, invasion, and metastasis of cancer cells. It can also cause non-activated CAFs to convert to a CAF-like phenotype. In addition, the stromal ECM or fibroblasts can modulate the immune cell population in the tumor microenvironment by preventing the infiltration of effector cells and expressing PD-L1 73. Due to these interactions between the tumor and its stroma, it is widely recognized that a single monotherapy cannot provide cure to cancer with certainty, and repetitive monotherapies run the risk of cancer cells developing resistance (**Figure 2A**). In addition, the hypoxic tumor microenvironment can lower the efficacy of radiotherapy or chemotherapy and contribute to the development of resistance. However, low pH or oxygen environment sensitizes tumor and endothelial cells to freezing 74. Oxygen level after the initial cryoablation recovers within hours and after 1 to 3 days, the oxygen level within the whole tumor tissue and particularly the periphery of the tumor increases drastically, setting up an ideal environment for subsequent radiotherapy or chemotherapy. Hence, there is a case for combination therapies with cryoablation to completely eradicate tumor cells without recurrence. There have been recent successful examples of combination therapy with cryoablation and other treatments such as chemotherapy, radiotherapy, surgery, embolization, and immunotherapy (**Figure 2B**). Clinical benefits of those cryo-based combination therapies are highlighted in **Table 2**.

### Chemotherapy

Since Jerrel W. Benson reported the pilot work of the combination therapy of cryoablation and chemotherapy for treating advanced oral carcinomas in 1975 107, various chemicals, such as ethanol 108, peplomycin, 5-fluorouracil, bleomycin, and doxorubicin, have been screened to combine with cryoablation to improve the treatment of prostate, lung, renal, and liver tumor in both animal tumor models and human clinical applications. Drugs that inhibit protein kinases have proven to be effective in greater selective killing of cancer cells. One such example is gefitinib, which is an epidermal growth factor receptor-tyrosine kinase (EGFR) inhibitor used to treat advanced non-small cell lung cancer (NSCLC) by interrupting signaling through the EGFR in target cells. Its specificity is higher than conventional chemotherapy and has less cytotoxicity. However, its clinical outcome of anticancer activity is only around 20-30%. Thus, it may see maximum benefit when used in combination with other therapies. The curative effect of cryoablation was heightened when combined with gefitinib in patients with advanced non-small cell lung cancer 81. Cryoablation combined with sorafenib had superior clinical efficacy compared with sorafenib-only for the treatment of advanced renal cell carcinoma unsuitable for surgical treatment. Moreover, this combined therapy induced anti-tumor immunological response and significantly prolonged progression-free survival time (PFS) and overall survival (OS) without compromising patient quality of life 77, 78.

Conventional systemic chemotherapy is similarly able to improve therapeutical efficacy when combined with cryoablation. Using a xenogenic mouse model, Le Pivert et al. were able to enhance growth inhibition of human prostrate tumors with the addition of 5-fluorouracil to cryoablation. The combination therapy overcame one of the limitations of cryoablation monotherapy - low efficacy due to partial freezing of tissue, usually occurring at the edge of the ice ball generated during cryoablation 79. Percutaneous cryoablation combined with systemic chemotherapy in the treatment of liver metastases from esophageal carcinoma is safe and also effective in improving the patients' quality of life 109, while in patients with liver metastasis from colorectal cancer, it improves the overall survival and tumor shrinkage rate (62.5 vs 22.6%) compared to chemotherapy alone 110. Even though the primary purpose of combination therapies is to enhance cancer cytotoxicity, certain chemotherapeutic agents may be added to cryoablation to improve secondary clinical outcomes, such as quality of life or pain levels. Interestingly, combination of zoledronic acid with cryoablation results in greater pain reduction and longer duration of pain alleviation than either therapy alone 75, 76. Another method of chemotherapy is interstitial injection of epirubicin-ioversol-ethanol solution 111, which has been demonstrated to be a feasible addition to cryoablation. While systemic chemotherapy can provide additional benefits to cryoablation, it traditionally results in undesired side effects. To circumvent this issue, there have been efforts to minimize drug exposure to healthy tissues by modulating its release kinetics. The potential advantages of controlled drug release chemotherapy with cryoablation were reported by Le Pivert et al. Specifically, microencapsulated 5-fluorouracil maximized cumulative effect of sustained release of 5-fluorouracil (5FU) during a 21-day period and had greater local effectiveness of cryo-chemotherapy compared to systemic chemotherapy or cryoablation alone, including higher tumor growth inhibition and tumor necrosis rate 80. Their results suggest that cryo-chemotherapy may become an adjuvant or an alternative to palliative therapies. With progress in controlled, sustained drug release, synergistic therapies integrating advanced local drug delivery and cryoablation may become a viable option in clinics soon.

### Radiotherapy

Radiotherapy causes irreversible damage to the DNA of tumor cells in the irradiation field and promotes the release of tumor-related antigens, increases the production of cytokines, alters the tumor microenvironment, and activates the body's immune system to initiate an anti-tumor immune response. Further, ionizing radiation can penetrate deeply into the body and provide a complete, uniform eradication of tumor mass. Hence, by combining radiotherapy with cryoablation, the general disadvantage of ablative therapies in producing heterogeneous tumor destruction can be addressed. Radiotherapy can be categorized into two types: external or internal radiation. Typically, the external radiotherapy involves a beam of ionizing radiation delivered from outside the body to a localized area around the tumor for destruction. Five cases of advanced-stage hepatic malignancies treated from 2017-2018 indicated that cryoablation combined with external intensity-modulated radiotherapy can be implemented safely. No complications above grade II occurred in the five patients, and their quality of life was significantly improved. Four patients experienced no disease progression and longer survival, with three of them still alive at the time of the study 97. Different from the previous type of radiotherapy, brachytherapy is a type of internal radiation therapy used to treat cancer. In this case, radioactive material is placed near tumors inside the patient, allowing higher, localized dose of radiation in less time, and reducing systemic exposure to ionizing radiation. Niu et al. found that cryoablation combined with iodine-125 seed implantations could be a palliative treatment of cardiac metastasis from alveolar soft part sarcoma 99. Results reported by the same group proved percutaneous combination treatment of iodine-125 and cryoablation may have a useful role in the management of stage IV pancreatic cancer less than 6 cm in diameter when surgery and chemotherapy are not options 100, 101.

Aside from direct first-line therapies, cryoablation is also used as a second-line therapy after radiotherapy to treat the tumor recurrence. Biochemical recurrence (rise in prostate-specific antigen level in blood) of prostate cancer after a form of radiotherapy occurs in approximately 26%-52% of patients. In cases of tumor recurrence following the biochemical failure, cryoablation may be applied to patients as a second-line, salvage therapy 63. Evidence points to greater survival in patients treated with cryoablation after radiotherapy. A single-center, retrospective experience with 32 patients confirmed that cryoablation offers a safe and effective alternative for locally recurrent prostate cancer with 100 % of 5-year cancer-specific survival and 43.5% of 5-year biochemical recurrence-free survival after radiation therapy 94. Another prospective study detailing long-term clinical outcomes showed that cryoablation is a viable salvage option for radio-recurrent prostate cancer, providing five and ten-year overall survival rate at 93% and 76%, respectively, and biochemical disease-free survival of 35% at ten years and 22.6% in 15 years 95. In a separate study, 23.1% of one hundred twenty-two patients who underwent salvage cryoablation had a positive biopsy for prostate cancer after a mean follow-up of 56 months 96. Despite the good overall survival rates, it is worth noting that tumor recurrence in the prostate continues to be a problem. These findings indicate that current strategies need improvement to improve outcome of salvage cryoablation. Lastly, tumor oxygen level is a crucial factor influencing clinical outcome after radiotherapy. Hypoxic conditions reduce the efficacy of radiotherapy, resulting in resistant tumor cells and lower overall survival of patients. Because cryoablation can transiently increase the oxygen level from hours to days after the treatment, it may be beneficial to pair radiotherapy after cryoablation. It is unclear whether the order of treatments impact therapeutic efficacy of the combination therapy or patient outcomes. Comparative studies systematically reviewing the clinical outcomes will help strategize the combination of cryoablation and radiotherapy.

### Surgery

Although cryoablation is usually a preferred option in patients with unresectable cancer, it can be combined with surgery in certain cases where there is a need to achieve a complete response. In a pilot experiment, twenty participants with breast cancer (<15 mm) underwent ultrasound-guided cryoablation before subsequent surgical resection. Here, cryoablation was used to combine with traditional surgical excision to assess the diagnosis effectiveness of contrast-enhanced MRI and improve therapy outcome by eradicating residual cancer post cryoablation 68. In addition to primary goal of eradicating tumor, surgery may be combined with cryoablation for patient comfort and to reduce complications. Palliative bypass surgery is sometimes done on patients with unresectable pancreatic cancer to direct bile flow into the small intestine. Despite not treating the tumor itself, it can provide pain relief. However, because the tumor growth is not treated, patients may experience increasing and debilitating pain. Cryosurgery combined with palliative bypass surgery can enhance quality of life, as shown in 74 patients with improved median survival from 8.5 months in the palliative bypass surgery only group to 14.5 months 91. Despite no significant improvements in 5-year overall survival rate, this approach can be considered a safe palliative treatment for unresectable pancreatic cancer. Cryoablation is also effective in reducing instances of post-operative bleeding (from 6-8% to 3.4%) during a maze procedure for atrial fibrillation 92. A combination of laparoscopic surgery and cryoablation was shown to be feasible in select patients to treat multi-visceral tumors, although further work is needed to test its viability in a wider patient range 90.

### Embolization

Transcatheter arterial chemoembolization (TACE), local treatment of HCC, is considered to have fewer side effects compared with systemic chemotherapy 112. Given that obstructing the blood flow from the hepatic artery to vessels around the tumor slows heat transfer, TACE may have synergy with cryoablation. A systemic review of patients with unresectable HCCs showed that those who were treated with a combination of transcatheter arterial chemoembolization (TACE) and cryoablation had better survival rate, complete necrosis, and less tumor recurrence compared to the TACE-only treated patients 113. Multiple studies have concluded that cryoablation combined with TACE is effective and safe in the treatment of advanced HCC 103, 104. However, there are still many factors that impact the treatment effectiveness, such as age, tumor diameter, tumor periportal location, and grade of liver function (Child-Pugh). Another factor that may need to be taken account in practice is order of treatment. It has been reported that hypoxic or acidic tumor conditions can sensitize the cancer cells to freezing 74. Hence, pre-conditioning of the tumor microenvironment by embolization to create hypoxic environment may enhance the efficacy of the follow-up cryoablation. A study enrolling 427 patients with unresectable HCC indicated that pre-cryosurgical transarterial chemoembolization (TACE) can increase the cryoablation efficacy and decrease its adverse effects, especially bleeding. Sequential TACE then cryosurgery may be the better procedure for unresectable HCC, especially for large HCC 102.

### Immunotherapy

#### Cryoablation-induced Immune Response

It is conjectured that cryoablation itself elicits immune response. Cryoablation-mediated necrosis results in immune response via recognition of the released intracellular contents by activated T cells. This immune response is not shown in apoptotic cell death, which resolves into smaller apoptotic bodies without breaking the cell membrane and thus exposing the signaling molecules that involve immune response 114, 115. The immune response includes a maturing of dendritic cells (DCs) to activate T cells, or increased levels of IL-6, IL-10, and TNF-α in serum 116. It has been shown that prostate cancer patients demonstrate a significant increase in tumor-infiltrating CD8^+^ T cells and decrease in CD4^+^ T cells near cryo-ablated tissues compared to non-ablated tissues 117. In addition, while renal arterial embolization by Lipiodol does not result in significant immune response, the combination of embolization and cryoablation shows a decrease in the percentages of Treg cells and increase in the percentages of CD4^+^ and CD4^+^/CD8^+^ T cells 105.

#### Cryoablation with Immunotherapy

**Figure 3:**
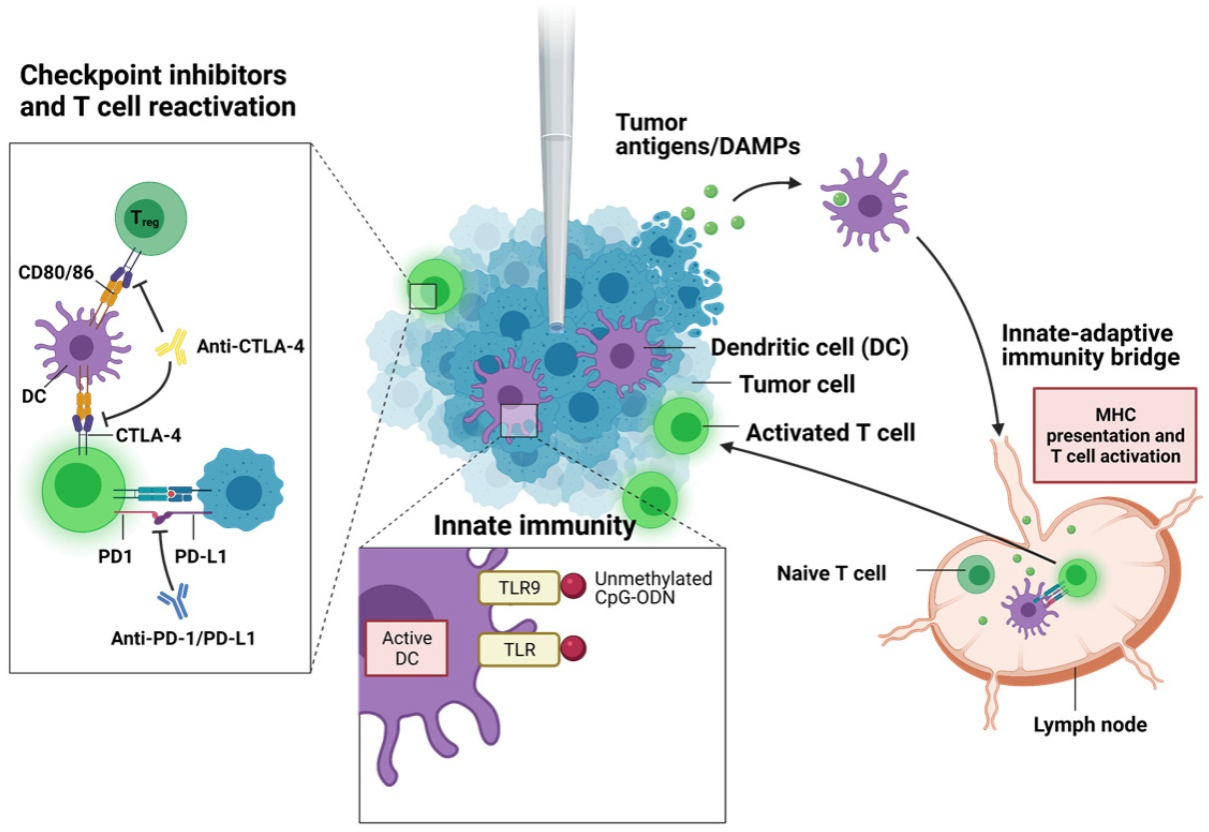
** Immunotherapeutic targets combined with cryoablation.** After the initial necrotic death by the freeze-thaw cycle of cryoablation, cellular contents including tumor antigens and damage-associated molecular patterns (DAMPs) are digested and processed by professional antigen-presenting cells such as dendritic cells (DCs). DCs can present the endogenous tumor antigens with major histocompatibility complex (MHC) class I molecules and MHC class II molecules (via cross-presentation). Immunostimulants that bind to toll-like receptors (TLRs) may activate and recruit DCs. DCs can then travel to lymph nodes, where naïve T cells will bind to antigen-presenting DCs through T cell receptors and costimulatory complexes such as CD28-CD80/86. Activated T cells can then circulate throughout the body and reach the tumor site, where they can carry out their cytotoxic function. In some cancer types, tumor cells may express programmed cell death-ligand 1 (PD-L1) that binds to programmed cell death protein 1 (PD-1) expressed on activated T cells, allowing T cell suppression. In addition, T cells may present inhibitory receptor cytotoxic T-lymphocyte-associated protein 4 (CTLA-4) that further downregulates antitumor response. Immune checkpoint inhibitors (ICIs) bind to these ligands and receptors and interfere with immunosuppression.

To enhance the innate immunogenicity of the cryoablation, several immunotherapies involving adoptive cell transfer, immune checkpoint inhibitors, and immunostimulants can be combined synergistically with the ablative therapy (**Figure 3**) 3, 118. Discovery of immune checkpoint inhibitors (ICI) were considered a breakthrough in immunotherapy and have garnered much attention in clinical trials in the recent years. Two major ICIs, anti-programmed death protein-1 (anti-PD-1) or anti-programmed death ligand (anti-PD-L1) and anti-cytotoxic T-lymphocyte-associated protein 4 (anti-CTLA-4), are currently approved to treat many cancer types, including solid tumors. Certain kinds of tumor cells express PD-L1 that interacts with PD-1 on T cells to suppress the T cell function and proliferation. PD-1/PD-L1 inhibitor blocks PD-1 or PD-L1 activity, allowing the T cells to recognize and attack cancer cells. Anti-CTLA-4 treatment works by blocking inhibitory receptor CTLA-4 on conventional T cells and regulatory T cells (T_reg_). Normally, T cell activation requires costimulatory signals from antigen presenting cells (APCs) aside from antigen-specific T cell receptor engagement. CTLA-4 on effector T cells binds to CD80/86 on APCs, suppressing T cell activation and causing clonal anergy. Further, CTLA-4 is constitutively expressed on T_reg_ cells (unlike effector T cells) and aid in immunosuppressive functions of T_reg_ cells on dendritic cells. CTLA-4 blockers prevent these inhibitions and allow cytotoxic T cells to kill cancer cells. Despite early clinical success in ICIs, they alone are often not enough to sufficiently clear cancer cells. Recent studies have explored ICI treatments combined with other forms of cancer therapy including ablative options. Clinical work by Zhu et al. showed a significant reduction of metastatic lesions in patients with cervical carcinosarcoma when treated with a PD-1 blocker, pembrolizumab, following cryoablation 119. No lesions were detected on CT images at 1.5 months post administration of pembrolizumab, and it was remained for 7 months after the treatment. In another study, a patient with high tumor mutational burden metastatic cervical carcinoma saw an exceptional response to cryoablation followed by pembrolizumab 88. Cryoablation was also effective in achieving complete response for at least 9 months in another patient who had developed resistance to anti-PD-L1 and anti-CTLA-4 antibody immunotherapy 120. In mice, combination therapy of anti-CTLA-4 antibody and cryoablation was synergistic, slowing the distant tumor growth by 14.8 days (p = 0.0006) and decreasing the mortality rate by a factor of 4 (p = 0.0003) 121. Interestingly, combining PD-1 blockade with cryoablation in another group of mice in the same study did not show any improvement over either treatment alone in terms of improving survival or delaying tumor growth.

Next to T and B lymphocytes, natural killer (NK) cells are important components of the innate immune system and play a critical role in the early host defense against cancer. With progress in the NK cell biology field and in understanding NK function, adoptive NK cell transfer has developed to be a powerful cancer immunotherapy tool in various cancers treatment 122, 123. However, it is still limited in achieving complete response in clinic as monotherapy. To enhance its therapeutic potential, there are recent efforts to combine NK cell immunotherapy with other modes of cancer treatments 123. Cryoablation combined with multiple infusions of allogenic natural killer (NK) cells had synergistic effect on patients with HCC, reducing tumor biomarker alpha fetoprotein levels and improving median progression-free survival (PFS), tumor size reduction, and quality of life post-treatment 85. Similar clinical outcomes were seen in patients with NSCLC and RCC who saw better quality of life, disease control rate, and response rate and in cryo-NK group versus cryoablation alone 86, 124. Adoptive transfer of a special type of natural killer T cells, dendritic cell-activated cytokine-induced killer cells (DC-CIK), with cryoablation also significantly increased overall survival (OS) in metastatic HCC patients. Multiple cryo-immunotherapy was associated with a better prognosis than single cryo-immunotherapy 89.

Aside from immune checkpoint inhibitors, there are other class of immunostimulants that can activate the immune system. Certain immunostimulants bind to pattern recognition receptors such as toll-like receptors (TLR), C-type lectin receptors, NOD-like receptors, and RIG-I-like receptors, which then trigger pro-inflammatory pathways and initiate adaptive immune response with antigen-presenting dendritic cells. For instance, toll-like receptor 9 (TLR9) can recognize viral or bacterial DNA and can induce pro-inflammatory cytokines. Unmethylated single-strand DNA called cytidyl guanosyl oligodeoxynucleotide (CpG-ODN) is one such molecule recognized by TLR9 on B cells and plasmacytoid dendritic cells. Therapeutic efficacy was closely associated with immune-adjuvant CpG-ODN administration in the combined therapeutic protocol of cryoablation, dendritic cells (DCs), and CpG-ODN. *In situ* administration of CpG-ODN 12 h after DC injection might be considered the optimum application 82. Granulocyte macrophage colony-stimulating factor (GM-CSF) is another major immunostimulant. In the body, it is secreted by the endothelium and immune cells. It binds to GM-CSF receptors present on myeloid progenitor cells, inducing proliferation and differentiation into granulocytes including neutrophils. In a total of 12 patients, combined cryoablation with granulocyte macrophage colony-stimulating factor (GM-CSF) treatment was suggested to be an alternative treatment for metastatic hormone refractory prostate cancer and could induce tumor-specific T cell responses 83. A combined therapy of cryoablation and GM-CSF in mice led to an increase in the number and activated percent of DC in spleen, greater tumor-specific cytotoxic T lymphocyte function, and lower lung metastasis rate 84. Other immunostimulants include a vast array of polysaccharides, hormones, and bile acids. In a pre-clinical mice model, the value of combining cryoablation with an administration of protein-polysaccharide preparation (Krestin) in the prevention of growth of residual tumors was investigated 87. Besides reducing tumor growths, the combination therapy suppressed IL-4 and IL-10 production and marginally improved NK cell and cytotoxic T cell counts in splenocytes. Whether each one of these immunostimulants will act beneficially to cryoablation in patients must be examined further in the future.

## Image-Guided Cryoablation Cancer Therapy

Accurate preoperative staging including computed tomography (CT) scan and magnetic resonance imaging (MRI) with a paramagnetic contrast agent (e.g., gadolinium) is essential to define the extent and location of an internal disease and the involvement of major vessels and bile ducts. In this section, image-guided cryoprobe positioning and ice ball formation, as well as post-operative assessment, will be discussed. Three major imaging modalities, CT, MRI, and ultrasound (US) are compared in **Table 3**.

Image-guided cryoablation via multiple imaging modalities such as MRI 125, 126, CT 127-131, PET 132, and ultrasound (US) 133 is vital to the technical success of the procedure 60. Images can be observed to guide the cryoprobe to the tumor, for real-time monitoring of ice growth, and be evaluated by the researchers post-cryoablation to assess for residual unablated tumor and complications. This allows for larger and more precise zones of ablation compared to radiofrequency ablation or other heat-based ablations 17. The extent of ablation zone is often characterized to ensure minimal damage to nontumor organs adjacent to the target tissue 134. Different imaging modalities can be combined throughout the ablation procedure. For instance, Ma et al. used a combination of MR and CT or US to aid the cryoablation of liver tumors close to the surrounding structures, MR to plan the puncture route of the cryoprobe prior to the ablation, CT or US during the operation to determine the injection site, position of the patient (supine or prone), injection angle, and the amount of freezing 135. Image-guided cryoablation (pre-, intra-, and post-operative) improves technical success and complication rate 136, 137. Contrast agents are often used to pinpoint areas that are not well-visualized in unenhanced scans.

### Cryoprobe Placement

**Figure 4:**
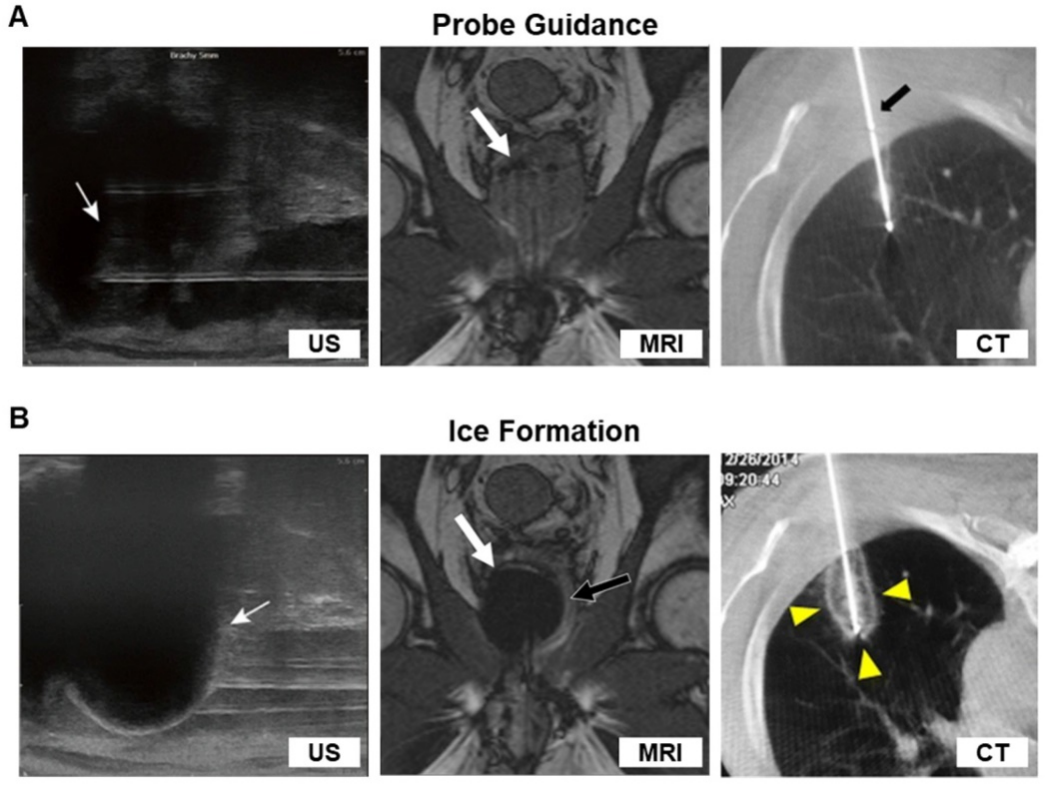
Pre-ablation guidance of the cryoprobes and real-time images of ice formation. (A) Ultrasound (US) image shows hyperechoic delineation of the probe and hypoechoic tumor (noted by arrows). In both the MR and CT image, the cryoprobe was brightly enhanced against the background. The arrows in the MR and CT images indicated the probes. (B) US image showed strong acoustic posterior shadowing by the ice ball. Likewise, T1-weighted MR image outlined the hypointense ice ball, and the CT scan showed ice ball formation (arrowhead) at the end of the cryoprobe. The US, MR, and CT images were adapted and reprinted under terms of the CC-BY license from ref. 162, 163, and 164, respectively. Copyright, 2017. Springer Nature. Copyright, 2019. The British Institute of Radiology. Copyright 2016, Springer Nature.

Pre-operative imaging is necessary for planning accurate placement of cryoprobes. Typically, the probe will be inserted through or parallel to the long axis of the tumor mass for the ice ball to form around the extent of the mass (**Figure 4A**). Among main imaging modalities, MRI, CT, PET, and ultrasound (US), only MRI, CT, and US can be used to position the cryoprobes.

#### Ultrasound (US)

A diagnostic ultrasound scan uses high-frequency sound waves emitted from a transducer which travel through the body and reflect back when they strike interfaces between regions of different densities. These echoes create images of the inside of the body. The cryoprobes are readily identified by their hyperechoic pattern with posterior acoustic shadow 67, 138. Technical aspects regarding feasibility and the safety of ultrasound imaging guided laparoscopic cryoablation procedure were addressed in a 1998 report of treating hepatic tumors 133. In summary, two principles can be followed to achieve the ideal working conditions of ultrasound imaging with cryoablation: (1) the ultrasound probe should be positioned on the tumor surface so that it shows the largest diameter of the whole lesion; (2) the ultrasound probe should be parallel to the cryoprobe. Reconstructing a three-dimensional vision of the cryoablation probes inside the lesion can be achieved by combining transverse and coronal/sagittal ultrasound image of the cryoprobe. In the study, ultrasound imaging was used to monitor the cryoprobe position at the margin of the tumor lesion and the treatment was successful with no major complications. Aside from liver cancer, ultrasound imaging guided cryoablation have been widely used in other cancer diseases, such as breast cancer 139, prostate cancer 140, renal cancer 141, and so on.

#### Computed Tomography (CT)

Computed tomography (CT) scanning, also known as computerized axial tomography scanning, is a diagnostic imaging procedure that uses x-rays to build cross-sectional images of the body. Cross-sections are reconstructed by calculating attenuation coefficients, or how easily the x-ray beams penetrate the volume of the object studied. Unlike ultrasound, CT offers a much better image with higher sensitivity, specificity, and accuracy, and it can be directed precisely at a target area, including but not limited to prostate cancer, pancreatic adenocarcinoma, and renal tumors 127, 136, 142, 143. However, ultrasound sees benefit from lower price, no radiation exposure, and real-time feedback. Further, for the diseases which are close to the skin, ultrasound provides a convenient method for positioning the cryoprobes. For imaging deep tissues, CT scan is preferred over US. Compared to US guided ablation procedure, CT-guided percutaneous cryoablation can be superior in treating lung tumors 144 because of the deeper detectability of CT. Non-contrast CT may be used to image tumors alongside cryoprobes, although it may become difficult to differentiate organs from similar-looking tumors. For instance, when the tumor in kidney resembles the renal parenchyma, it becomes difficult to visualize the location of the tumor 137. In this case, contrast-enhanced CT is needed to identify the tumor margin. Further, the choice of contrast agents should be deliberated. For example, using a high-concentration oil-based CT contrast agents during the image-guided cryoablation can limit the area of frozen region due to its low freezing point and weak thermal conductivity 145.

#### Magnetic Resonance Imaging (MRI)

In the 1990s, magnetic resonance imaging (MRI)-guided percutaneous procedures were initially performed in open-bore MRI units 146. Currently, the spectrum of interventional MRI-guided procedures has been extended to be routinely performed in both biopsies and ablations. In theory, closed-bore MRI with a relative high field (1.5 and 3-T) will allow higher quality images than open-bore MRI for cryoprobes position. Under the MR imaging system, cryoprobes can be placed in solid tumors and lesions ablated by using real-time monitoring 147. Like other noninvasive imaging modalities, MRI can be used to guide cryoablation in many tumor types, such as prostate cancer 148, hepatic cancer 149, and breast cancer 150. One study took advantage of MRI sensitivity to water by adding saline to displace rectal wall from prostate during MRI-guided cryoablation of prostate cancer, which produced clear margin around the rectal wall, preventing intrusion of ice ball formation into the rectum 125. Hence, the imaging modality may be considered depending on tumor type, location, and size. Although MR imaging has been reported to be superior to CT for cryoablation lesion detection and characterization, there are several downsides compared to other imaging methods. Disadvantages include limitation of operational space while involving close-bore MRI, need of MRI-compatible cryoprobes, near-real time movement of probes, the noise generated by MRI, longer time consumed in producing better images, and instances where MRI itself is contraindicated in patients.

### Real-time monitoring of ice formation

In general, a cryoablation procedure is deemed complete when imaging reveals ice-ball formation covering the entire tumor with a suitable margin (**Figure 4**). It is important for physicians to monitor the real-time formation of the ice ball so that the edge of the ice ball does not overextend into healthy tissues. This ability is a significant factor in giving cryoablation an advantage over other ablation therapies, because it allows for the killing of tumor cells while sparing surrounding tissues. Thus, early visualization of ice ball formation on ultrasound or CT is crucial since the necrotic isotherm tends to lag several millimeters behind the actual edge of the visible ice ball. In an *in vivo* porcine model, a triple-freeze protocol not only increased the area of cryolesion but also expanded the region of necrotic isotherm compared to a double-freeze protocol 151. Using modified triple-freeze protocols instead of double-freeze protocols is advantageous for earlier visualization of ice ball formation on CT 152. One study extended the CT imaging to dual-energy CT to visualize the real-time ice formation not only in soft tissues but also in skeletal structures 153. The dual-energy CT is beneficial in imaging bone metastases or nearby tumors obstructed bony tissue. In hepatic malignancies, a fusion of different imaging modalities such as pre-ablation MR and intraoperative CT can assess the ablative margin after cryoablation 154. MRI, like CT, can be used for real-time monitoring of cryoablation. Mala et al. used 0.5-T MRI to monitor the ice formation and measure the volume of cryolesion in patients with hepatic tumors; MRI provides good visualization of the growing cryolesion and shows optimal overlap of cryolesion and tumor area for tumors smaller than 3 cm 149. In another study, the 1-T open MRI-guided percutaneous cryoablation of hepatic dome HCCs could be successfully performed by real-time monitoring of the ice ball formation using a freehand MR fluoroscopy 155. Ultrasound (US), like other imaging modalities, provides intraprocedural monitoring, but is a cheaper alternative to MRI or CT 139. Like CT, the extent of necrosis due to ablation can be predicted consistently using US 156. However, ultrasound is operator dependent and its imaging quality may be influenced by several factors including patient habitus and abundant bowel gas 157. One limitation pertaining to the physics of ultrasound is that ultrasound is almost 100% reflected by an ice interface. Thus, frozen tissue cannot be imaged, and unfrozen tissue completely enveloped by frozen tissue also cannot be imaged. This presents a problem because posterior shadows by the edge of the ice ball may prevent the operator from seeing what part of the tumor has been treated (**Figure 4B**) 158. Hence, ultrasound likely presents more technical challenges than MRI and CT during intraoperative imaging.

While not commonly used in clinic practice for cryotherapy, near infrared (NIR) fluorescence imaging and NIR thermography have been explored in preclinical studies. Near infrared fluorescence imaging offers high spatial resolution, quick response rate, low cost, and portability. However, NIR fluorescence imaging suffers from limitations such as limited tissue penetration, tissue autofluorescence, and photobleaching 159. Further, it requires an imaging probe to differentiate tumor from other parts of the body, which adds toxicity.

Conventional temperature monitoring methods require the placement of thermocouples next to cryoprobes. However, they are invasive and can only measure the temperature of the cryoprobes and immediate vicinity, but not peripheral tissue temperatures. MR thermography opens opportunities for a non-invasive way to measure tissue temperature with high spatial and temporal resolution. It can be used to map the temperature gradient and track the progress of the cold front approaching the boundary between tumor margins and healthy tissues. Real-time temperature monitoring with thermography should be coupled with other imaging modalities to guide cryoablation. Multifunctional MR imaging, for example, can provide detailed anatomical information before and after cryoablation, but also accurate temperature monitoring and thus on-the-fly thermal regulation 160. NIR thermography can also measure temperature difference from a shift and narrowing in NIR spectral peaks due to temperature-dependent changes in intermolecular hydrogen bonding in water 161. It is still limited by low penetration of NIR waves, and background noises from tissue in front of the cryoprobes can introduce errors in measurement, making it difficult to accurately visualize ice ball growth in inner organs. Nevertheless, NIR thermography is non-invasive, quick, and small-scale, so there is merit in exploring it as an option for implementation in cryotherapy. There needs to be a heavy amount of data gathered, especially regarding temperature sensitivity in extremely low temperatures, to support *in vivo* applications.

### Follow-Up Assessment Post-Cryoablation

Use of radiotracers in cryoablation dates back more than 20 years, when it was observed that soft tissue uptake of technetium-99m-methylene diphosphonate (^99m^Tc-MDP) within the prostate bed was enhanced after cryoablation of prostate carcinoma 165. The post-cryoablated prostate bed was characterized by a liquefactive necrosis and increased blood flow and calcium concentration, which likely were reasons for the ^99m^Tc-MDP uptake. Whereas ^99m^Tc-MDP is now used mostly for single photon emission computed tomography (SPECT) of bone, one of the most common radiopharmaceuticals used today for tumor contrast is fluorodeoxyglucose (^18^F-FDG). In CT guided cryoablation of hepatic tumors, follow-up ^18^F-FDG PET/CT achieved 100% technical success and efficacy at 3 months post-cryoablation, superior to CT or MR imaging 26. Further, ^18^F-FDG PET-CT for the follow-up of small renal mass and adrenal metastasis cryoablation showed that ^18^F-FDG PET-CT imaging could be used to characterize cryoablative tissue injury at different times after cryoablation 132, 166. Beside ^18^F-FDG, other radionuclides may be used in PET. A report of one patient with clinical diagnosis of tumor-induced osteomalacia visualized with Ga-68 DOTATOC PET/CT and MRI showed successful application of PET/CT to locate and perform image-guided biopsy and cryoablation of a radiographically occult phosphaturic mesenchymal tumor mixed connective tissue 167. In some cases, PET or PET/CT have been considered as the standard for staging and surveillance of recurrent and metastatic disease 168, 169. It is expected that the inclusion of PET imaging in cryoablation will allow more sensitive and accurate evaluation post-treatment.

Post-treatment CT and MR imaging findings are crucial to assess the cryoablation efficiency. They have been extensively used in clinical practice mainly for detection and monitoring of cancer after treatment, particularly early-stage local tumor recurrences. Because PET/CT often have limited spatial resolution, they may have a poor time detecting small tumor lesions. Hence, the use of multiparametric MR imaging to detect recurrence at an early stage is becoming an important application of imaging modes in post-treatment of cancer 170. In one study, authors described the imaging appearance of renal cell carcinoma post-cryoablation using T1WI, T2WI, and diffusion-weighted imaging (DWI) scans 171. The ablated tumors displayed variable signal in T2WI and high signal in DWI for the first 3 months after the procedure. At 6 months, the tumors became hypointense in both T2WI and DWI and persisted afterwards. In T1WI, the ablated tumors maintained high signal intensity in the first 1 to 9 months and were prominently rim shaped. Tumor recurrence during this period was characterized with isointense or hypointense T1 signal coming from the margins or the insides of the tumors. Tumor recurrence could also be detected in T2WI when there was an interruption in the dense hypointense signal around the rims of the ablated tumors. CT is also a well-established imaging method to assess the ablation lesion and evaluate the treatment response. Compared to MRI, it costs less time to image the target site. Non-contrast-enhanced CT could also be used to evaluate the prognosis of tumor and to monitor local tumor recurrence like MRI. Chaudhry et al. were able to detect cavitation in the cryoablation zone which is an indicator for tumor progression, and measure signal intensity in the lung nodules to detect complete ablation (decreased intensity) or recurrence (increasing intensity) 172. In cases where contrast administration becomes unviable in patients, non-contrast-enhanced MRI may be preferred over non-contrast-enhanced CT, as the former has imaging sequences that allow differentiation of tissue characteristics.

## Nanomaterials in Cryoablation: A New Opportunity

Traditional disadvantages of ablation technologies have been incomplete destruction of tumor tissues and restriction on the treatable tumor size. Despite its ease of use and efficiency, cryoablation still faces problems from inadequate ice formation, possibility of the ice ball overextending into healthy tissues, and reduced tumor cell death on the periphery of the ablation zone. Due to these factors, recurrence can occur from the residual tumors treated with cryoablation. Combining chemotherapy or immunotherapy with ablation can address these issues by enhancing tumor cell death. However, the greater anticancer effect of combination therapies is concomitant with systemic side effects. Hence, local approach to adjuvant therapies with image-guided cryoablation is needed for precision targeting of the tumors. Further, it would be important to visualize co-localization of drugs with the cryolesion zone to ensure that the correct dosage is delivered. Ideally, the distribution of the drugs should remain within the ablated tumor site. Recent advances in biomaterials, especially nanoparticles, have shown the possibility of reaching these goals through imaging and local delivery of therapeutics. Pre-clinical models show great promise in these areas, although much work is needed to translate the results into clinical phases.

Nanomaterials can act as both imaging contrast agents and vehicles of chemotherapeutic or immunotherapeutic drugs 173. These two functions are key to image-guided nanomedicine in achieving highly localized drug in tumor sites with minimized side effects and tracking the drug biodistribution non-invasively for location and quantification. Some nanoparticles may generate reactive oxygen species (ROS) from radiation 174-177 or heat from light 178, further increasing antitumor effect. Given that ablation therapies rely heavily on imaging and often suffer from inadequate elimination of tumor cells, nanoparticles composed of inorganic metals or organic polymers and lipids can provide a platform for image-guided combination therapies. Particularly with the advent of immune checkpoint inhibitors, advanced MR, CT, SPECT, and PET image-guided delivery of multifunctional nanoparticles for the immuno-combination treatment can achieve higher therapeutic efficacy compared to conventional monotherapies such as ablation therapies and radiotherapies. In 2020, a novel approach combining immunotherapy with irreversible electroporation ablation reported magnetic iron oxide nanoclusters that vibrated upon an application of an electric field to release indoleamine-2,3 dioxygenase inhibitor for a synergistic immuno-ablation of tumors 179. MRI-guided, local, sustained release of anti-PD-L1 could also be achieved by capping of mesoporous silica particles with ferumoxytol or metallic nanoparticles 180, 181.

**Figure 5:**
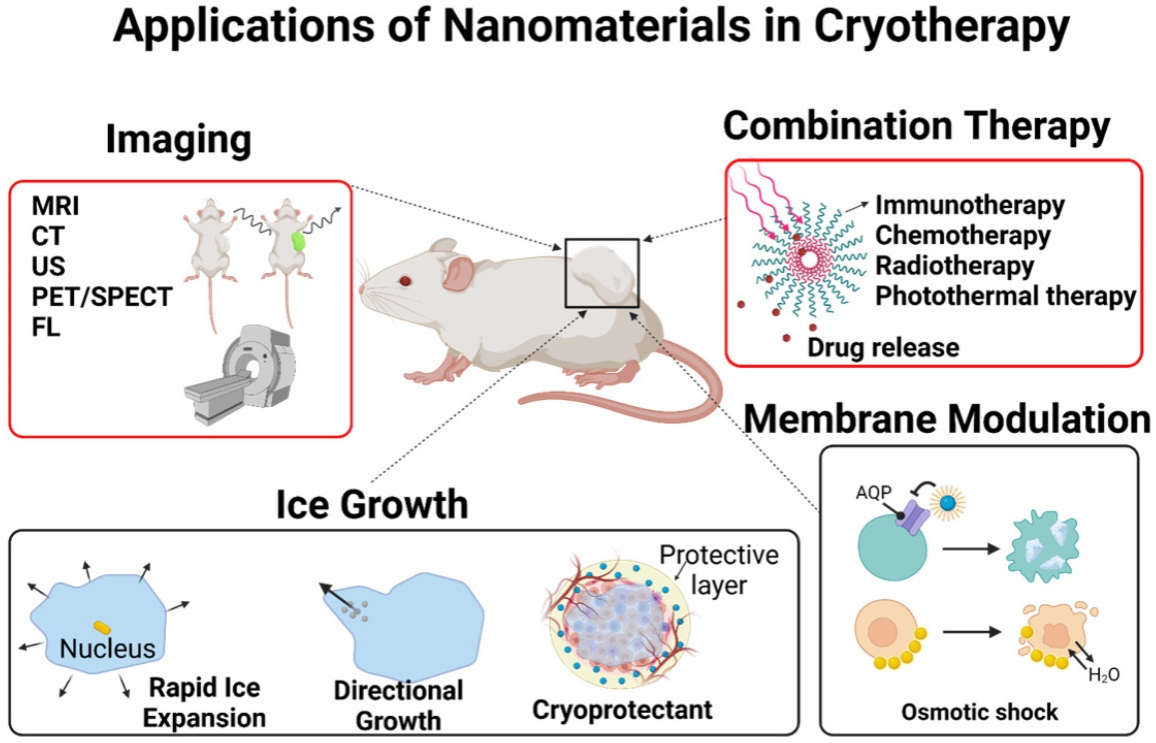
** Applications of nanomaterials in cryotherapy.** Nanoparticles can increase and direct ice growth, protect healthy tissues from cryoinjury, damage cells via modulating their membranes, locally release drugs, and enhance imaging. Image-guided local delivery of therapeutics via nanoparticles may minimize systemic toxicity while supporting sustained drug release in the tumor region. By tuning the composition of the nanoparticles, it is possible to induce ice formation in the tumors while insulating heat transfer to healthy tissues. Combined approach with nanoparticle-mediated cryoablation and other types of cancer therapy can be a promising direction in image-guided nanomedicine.

Although many examples of beneficial nanoparticle use in thermal ablation therapies have been discussed in the literature, not much have been seen in cryoablation therapies. The role of nanoparticles in cryoablation will be discussed in detail in this review. Briefly, nanoparticles can help cryoablation in the following possible manners: faster ice formation, inducing cellular damage by altering properties of cell membranes, controlled drug release in thaw-freeze cycles, enhanced imaging, controlling ice shape, and protecting surrounding healthy tissues from cryoinjury (**Figure 5**). The nanoparticles useful in cryoablation can be divided into four general categories: metal, organic (polymeric, lipid-based), liquid metal, and hybrid of the three previous materials. **Table 4** briefly summarizes the nanoparticles discussed below.

### Catalysis for Ice Formation and Osmotic Stress

There are several factors to consider when designing nanoparticles to either enhance or hinder ice formation and cryotoxicity, namely their size, surface area, material composition, and surface properties. In freezing, ice nucleation is a controlling step. It is widely believed that critical ice nucleus is necessary for ice to nucleate. Bai et al. experimentally demonstrated that graphene oxide nanosheets smaller than the critical ice nucleus (~8 nm) suppresses ice formation, while those larger than the critical ice nucleus (>11 nm) promotes ice growth 182. Below 8 nm in size, the concentration and size of nanoparticles does not affect the ice nucleation temperature, equal to the temperature measured for water absent of the nanoparticles. There is a sharp rise in the ice nucleation temperature moving from a size of 8 nm to 11 nm. With diameters beyond 11 nm, the ice nucleation temperature of water droplets containing the graphene oxide nanoparticles increases, but not substantially. Hence, there appears to be a lower limit on the size of the nanomaterial which holds significance in the rate of ice growth. Closely related to size is the surface area. Surface area of the nanoparticles should also be considered when increasing ice formation. Surface area of the nanoparticles is directly correlated to heat transfer between the nanoparticles and the external surrounding environment. Hierarchical nanostructures such as branched nanoparticles or nanofibrils exhibit high surface area-to-volume ratio. Branched gold nanoparticles and flowery gold-silver bimetallic nanoparticles have been known to increase heat transfer in photothermal therapies 183-186. In a similar manner, these types of highly ordered and thermal nanostructures can rapidly cool cancerous tissues.

**Figure 6:**
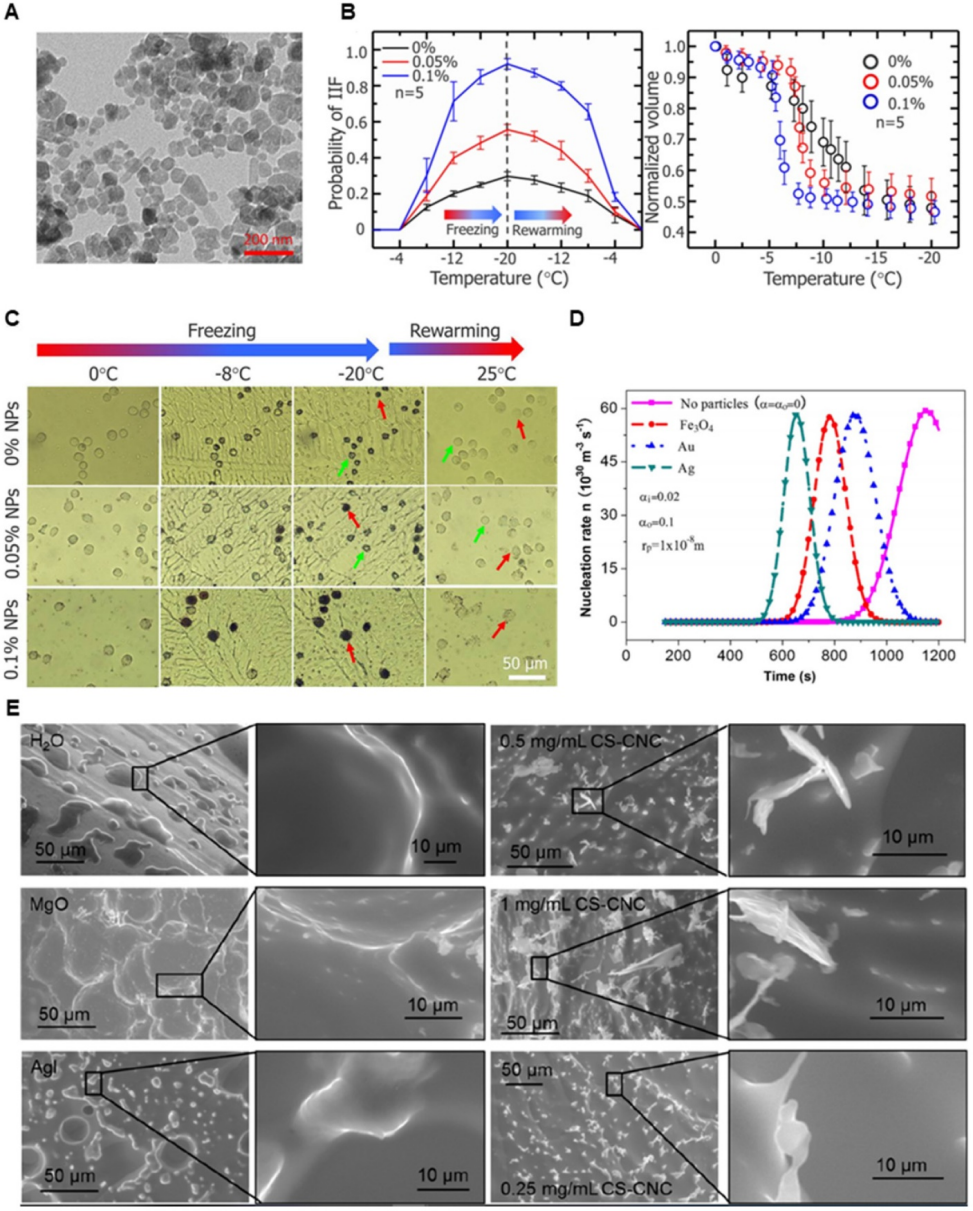
** Effect of different nanoparticles on intracellular ice formation. (A)** TEM image of Fe_3_O_4_ nanoparticles. **(B)** Probability of intracellular ice formation increased with increasing concentrations (0, 0.05, and 0.1% w/v) of Fe_3_O_4_ nanoparticles while the cell volume decreased. **(C)** Cryomicroscopic images of HepG2 cells treated with Fe_3_O_4_ nanoparticles showed formation of intracellular ice. Red arrows indicate intracellular ice and green arrows indicate no intracellular ice formation. Scale bar is 200 nm in (A) and 50 µm in (C). Adapted and reprinted from ref. 189 under terms of the CC-BY license. Copyright, 2017. Impact Journals. **(D)** Au, Ag, and Fe_3_O_4_ nanoparticles affected cell nucleation rate at various rates, with fastest intracellular ice formation in cells containing Ag nanoparticles. Adapted and reprinted with permission from ref. 190. Copyright, 2008. American Institute of Physics. **(E)** Cryo-SEM images demonstrated rounded edges of ice crystals formed in pure water and water dispersed with AgI or MgO nanoparticles and sharp edges in the CS-CNC group. Adapted and reprinted from ref. 198. Copyright 2021, American Chemical Society.

In terms of heat transfer, metal nanoparticles generally have higher thermal conductivity than polymeric or lipid-based nanoparticles and can therefore exchange heat more efficiently. They can permeate throughout the tumor and allow more uniform temperature gradient during cryoablation. Further, when the nanoparticles accumulate around the tumor tissues due to EPR effect and enter cells, they can enhance intracellular ice formation. MgO, gold, and silver nanoparticles have shown high thermal conductivity. Biodegradable MgO nanoparticles increases the rate of intracellular ice formation and can be used as an adjuvant to cryoablation without added risk of toxicity 187. Like MgO nanoparticles, gold (Au), silver (Ag), and Fe_3_O_4_ nanoparticles increase the rate of ice growth and intracellular ice formation by acting as nuclei for ice crystallization 188-190. Yuan et al. showed that Fe_3_O_4_ nanoparticles increases the probability of intracellular ice formation and cellular dehydration after the freezing and thawing cycle (**Figure 6A-B**) 189. The intracellular ice formation increases with greater concentration of the nanoparticles in the tumor (**Figure 6B-C**). Meanwhile, Au nanoparticles result in faster ice ball growth, but not earlier intracellular ice formation than Fe_3_O_4_ nanoparticles, suggesting that there are variances between metal nanoparticles (**Figure 6D**), likely due to differences in the rate of nanoparticle cellular uptake 190. Different composition, size, and shape will all likely influence the freezing effect of cryoablation. Given the ability of nanoparticles to enhance ice growth, directional manipulation of ice ball shape can be attained. Deposition of nanoparticles in tumors (aggregation at the cell membrane) may lead to the growth of an ice ball to match the irregular tumor shape. Directional freezing, or ice growth, is possible when injecting cryo-enhancing aqueous suspension of aluminum nanoparticles and cryoprotective dimethyl sulfoxide (DMSO) at different positions 192. Artificially controlling the ice growth to match the boundaries of tumors that are irregularly shaped may be feasible in real-time with image contrasting nanoparticles. Less thermally conductive non-metal nanoparticles may be used to lower the side effects from cryoablation. Some polymeric or lipid nanoparticles may be useful in protecting healthy tissues from cryoinjury due to their inefficient heat transfer. Liposomes loaded with phase change materials (PCMs) that have low thermal conductivity and high latent heat may significantly reduce cryoinjury 193. Chitosan-tripolyphosphate (CS-TPP) nanoparticles loaded with cryoprotectant trehalose are also able to preserve NK cells from cryoinjury without additional need of cytotoxic cryoprotectants like DMSO 194. Recovery of CS-TPP-treated NK cells is superior to the conventional cryoprotectant, DMSO, (**Figure 8C**) and CS-TPP nanoparticles improve cellular penetration and raise the efficiency of trehalose. Preserving important immune cells in the surrounding the health tissues may benefit immune response post-cryoablation of the tumor.

Surface functional groups of the nanomaterials are important in altering the shape of ice crystals and crystallization growth. Hydrophobic interactions and hydrogen bonding between the surface functional groups and water molecules have been regarded as main attributes for preferential binding of synthetic nanomaterials or natural antifreeze proteins to ice crystals to depress crystallization 191, 195, 196. Depending on the placement of the functional groups ice growth can be enhanced or inhibited, resulting in directional shaping of the ice crystal. Shape of the nanomaterials can translationally affect the shape of ice crystals. Ice crystals which are sharp and long may be advantageous to cryoablation if the intracellular ice crystals can pierce into organelles or the cell membrane. For instance, it was recently reported that aqueous dispersions of poly(ethylene glycol)-poly(l-alanine) (PEG-PA) could form round ice crystals while poly(vinyl alcohol) (PVA) could form needle-like crystals 197. PEG-PA had high cryoprotection while PVA did not. Similarly, chitosan-coated cellulose nanocrystals (CS-CNC) could facilitate needle-like ice crystal growth, intracellular ice formation, and faster ice nucleation rate 198. Compared to MgO and AgI nanoparticles, CS-CNC nanorods produced sharper ice crystal edges (**Figure 6E**). The effect is likely due to increased hydrogen bonding between water molecules and the carboxyl groups of cellulose at a specific surface of the nanorods. Therefore, surface modification and shape of nanoparticles are facets to examine for cryo-cytotoxic or cryoprotective effects. However, there needs to be more studies done to evaluate the damaging effect of ice crystal morphology, as it is not clear how much of cryotoxicity is credited to the geometry of the ice crystals. Further, these nanoparticles must be studied under a variety of animal models to prove their efficacy in a physiological environment which contains many solutes and biological molecules that can affect therapeutic outcomes.

Another mechanism of cryoinjury is osmotic stress. As ice accumulates in the extracellular space, concentration of solutes will increase, thus prompting water to rush out of cells. The resulting cellular dehydration can be potentially lethal. When there are intracellular ice crystals, the osmotic gradient across the cell membrane is maintained and dehydration is less severe. Whether the formation of intracellular ice crystals or dehydration that dominates cellular death is still elusive. Regardless, membrane fluidity and permeability are crucial to cell viability during the freeze-thaw process 199. More fluid cells tend to survive due to flexibility in the membranes as they swell or shrink under osmotic pressure. Rigid membranes suffer breakage under the cold temperatures as water rushes in and out of the cells. Further, permeabilizing the membranes can ease osmotic stress from damaging the cells and reduce the chance of intracellular ice crystals forming from water inside the cells. Cryoprotectants like DMSO or amphiphilic molecules work with the same principle. Therefore, controlling the biomechanics of cell membranes with nanoparticles is a rational approach to increasing cellular injury during cryotherapy. Within the body, when cells are subjected to a freezing temperature, they form additional aquaporins as an adaptive mechanism to osmotic stress and intracellular ice formation 200. Many types of cancer, including cancer of prostate, breast, liver, renal, and skin, incidentally, overexpress aquaporins as a mechanism for tumor migration, invasion, and metastasis. Aquaporins have also been correlated with poor prognosis of patients in a number of cancers 201. However, as aquaporins exist in normal cells as well, nanoparticle-based delivery system of aquaporin inhibitors can be used to reduce the chance of side effects and synergize with cryoablation. Inhibition of aquaporins in cultured prostate and breast cancer cells enhanced cryoinjury 199, 200.

Physical binding of the cell membrane by nanoparticles can also change its fluidity and permeability and sensitize the cells to cryo-shock. Nanoparticles of certain sizes and charge can adsorb onto the cell membranes and perturb their mechanical properties. Citrate-capped anionic gold nanoparticles of 13 nm in diameter were able to adsorb onto the lipid bilayer of liposomes and induce lipid gelation 202. Tight complexation of the citrate-gold nanoparticles with the zwitterionic phosphocholine head group of the lipids formed a structure akin to lipid rafts. Due to a drag force associated to their heavier weight and particle size, the nanoparticle-lipid complexes diffused more slowly than undecorated lipids, lowering the membrane fluidity. Anionic gold nanoparticles adsorbed onto the surface of a cell membrane could perturb the head group orientation of lipids and disrupt ion channels 203. In terms of membrane fluidity, size of nanoparticles is an important factor that affect the energy required for membrane wrapping around the nanoparticles. In silica nanoparticles, smaller nanoparticles (~18 nm) displayed “freezing” effect of the lipid membranes by adsorbing onto their surfaces and significantly reduced lateral mobility of phospholipids, whereas larger nanoparticles (182 nm) induced wrapping of the membranes and led to a moderate increase in fluidity 204. Further, hydrophilicity/hydrophobicity of the nanoparticles influenced the membrane permeability. Amphiphilic gold nanoparticles were able to embed themselves within the lipid bilayer and induce local permeability of the membrane 205, 206. Charge, size, and hydrophobicity of the nanoparticles will all play part in maintaining the integrity of the cell membrane after freezing and thawing. However, more studies are needed to investigate the effects of nanoparticle-biomembrane interactions under freezing conditions. At this time, it is unclear how much benefit the modulation of biophysical properties of membranes can affect cell viability after freezing and thawing.

### Imaging Contrast

**Figure 7:**
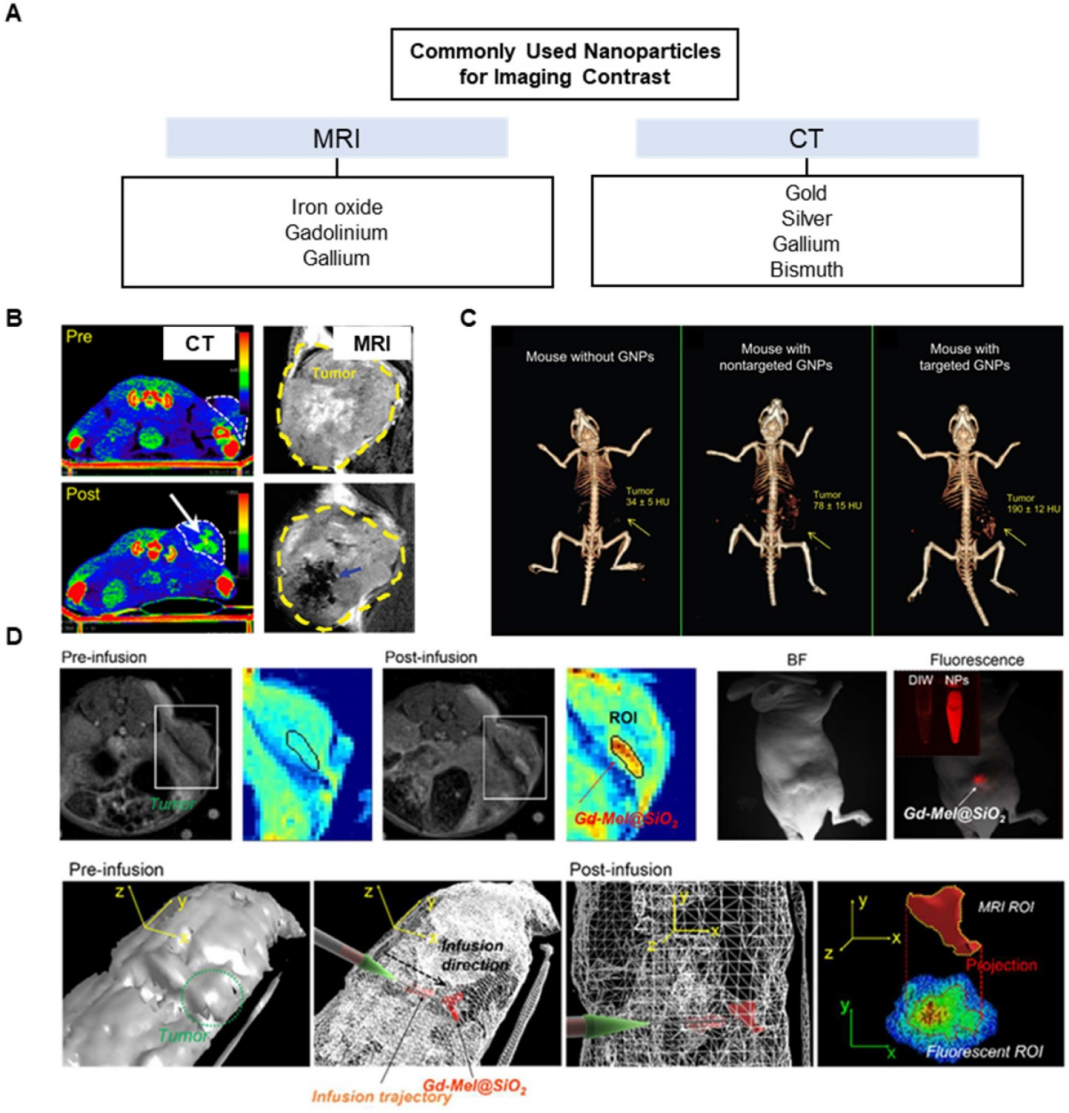
** Nanoparticles used to enhance MR/CT image contrast. (A)** Commonly used nanoparticles for MR and CT imaging. **(B)** Contrast-enhanced CT and MR images from gold nanoparticles and iron oxide nanoparticles, respectively. The nanoparticles are indicated by arrows. Adapted and reprinted with permission from ref. 176 and 180. Copyright 2018, American Chemical Society. Copyright 2019, Wiley-VCH. **(C)** *In vivo* CT volume-rendered images of mice before injection of gold nanoparticles (GNPs), 6 h post-injection of nonspecific immunoglobin G decorated GNPs, and 6 h post-injection of anti-EGFR-coated GNPs. **(D)** MR, fluorescence, and merged MR/fluorescence images of mice showed clear visualization of tumor after injection of silica-coated melanin-gadolinium nanoparticles. Adapted and reprinted with permission from ref. 216. Copyright 2017, American Chemical Society.

Various types of nanoparticles have been tested in the recent years for high imaging contrast during therapeutic procedures (**Figure 7A**). Because nanoparticles tend to remain in the tumor regions via EPR effect, they have a longer half-life over small molecule contrast agents. During cryoablation, the delineation of tumor edges is highly important in ensuring that the ice ball does not extend into healthy tissues. Thus, cryoablation can only be used for tumors that can be seen by imaging. In tumors that do not show clearly defined edges on images, it may be beneficial to use targeting nanoparticles to accumulate near the tumor edges. Typically, certain classes of metal nanoparticles can act as intrinsic imaging contrasts, while other types of organic nanoparticles may instead carry contrast agents such as gadolinium for MR or Lipiodol for CT. Currently, metal nanoparticles that do not need molecular probes or dyes avoid the problem of additional toxicity. Gold nanoparticles have been extensively used as CT contrast agent in preclinical and clinical settings 207-209. Silver nanoparticles have also been explored as contrast agents for CT imaging 176, 210, 211, while magnetic iron oxide nanoparticles are visualized with MRI 180, 212 (**Figure 7B**). Depending on a diagnostic biopsy, these imaging nanoparticles can be utilized to actively target tumors. Active targeting of tumor cells involves ligand-receptor interactions for specific homing. Nanoparticles may be decorated with ligands to target tumor receptors (e.g., HER2, folate, sigma-1, CD44) for increased affinity and specificity over passive targeting. Gold nanoparticles loaded with anti-epidermal growth factor receptor (anti-EGFR) have shown clear contrast in the tumor regions over passive targeted gold nanoparticles coated with nonspecific immunoglobin G antibodies 213 (**Figure 7C**).

**Figure 8:**
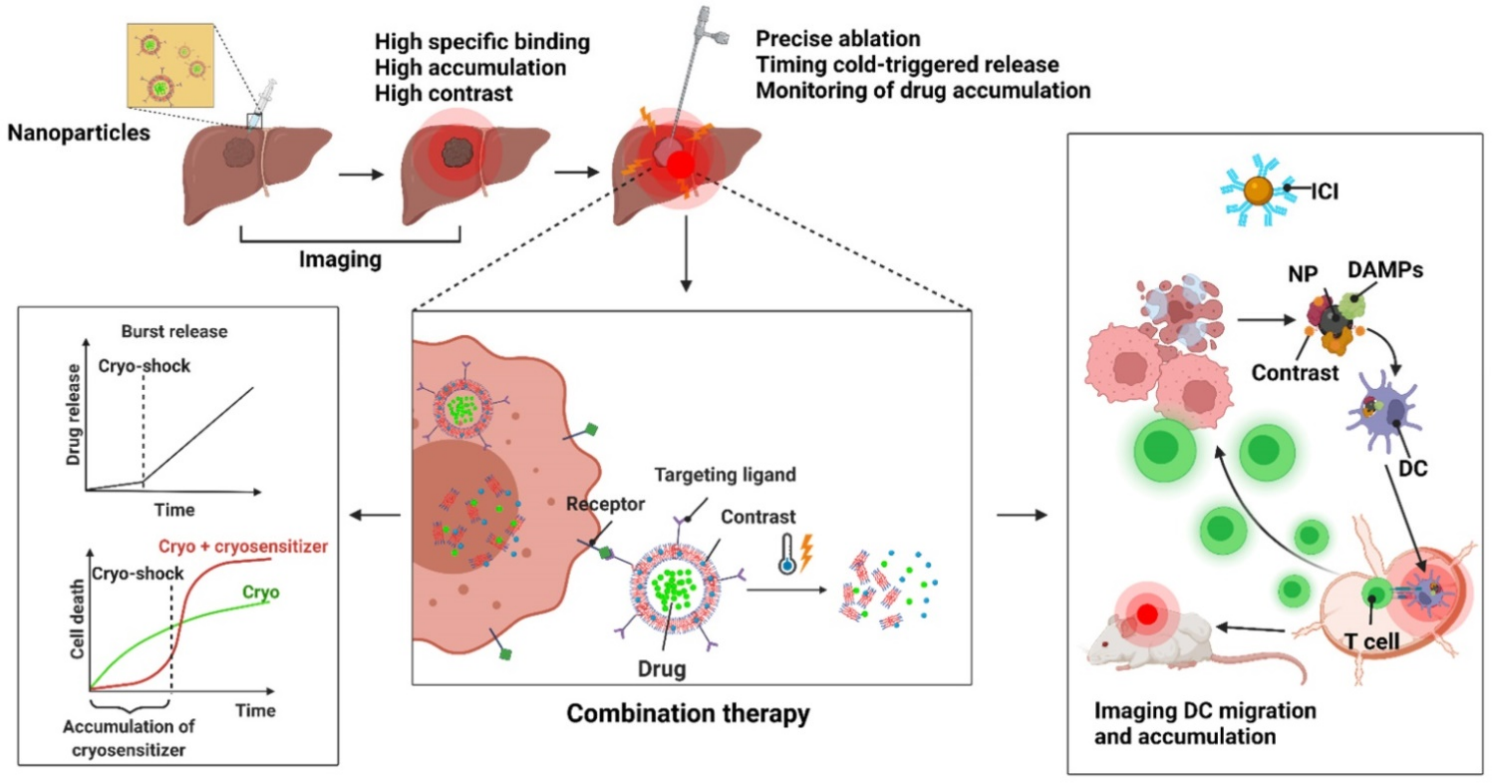
** Translational application of nanoparticles in combination cryotherapy.** Theranostics nanoparticles allow co-delivery of contrast agents and drugs to tumor for an accurate delineation of tumor margins and monitoring of drug distribution for more precise ablation and synergistic combination with cryotherapy. Nanoparticles may be imaged the accumulation in the tumor and tailored to trigger on demand with temperature drop from cryoablation to increase cell death and overcome drug resistance. Imaging can be used to confirm deposition of such nanoparticles in the tumor site before applying cold shock to rapidly increase the concentration of drugs in cancer cells or to maximize cell death from cryoablation with cryosensitizers. Imaging can additionally confirm the delivery of immunomodulators in the tumors or presentation of tumoral antigens carried by nanoparticles in the secondary lymphoid organs.

Bimetallic nanoparticles such as iron oxide-gold nanoparticles can provide both MR and CT contrast 214, 215, and hybrid nanoparticles loaded with contrasting agents such as silica-coated melanin nanoparticles carrying gadolinium and fluorescent dyes can be utilized for MR/fluorescence imaging 216 (**Figure 7D**). These types of multimodal imaging nanoparticles provide advantages of each imaging modality. MR imaging using gadolinium is suitable for assessing drug release from nanoparticles, since it relies on the movement of water molecules. Temperature-sensitive liposomes co-encapsulating gadolinium and doxorubicin demonstrated correlated release between doxorubicin and gadolinium 217. Gadolinium-conjugated drug or drug-conjugated iron oxide nanoparticles are also be used to delineate the biodistribution of drugs 218. Thus, a theranostic nanoparticle can be designed to monitor an accumulation of a cryosensitizing agents such as vitamin D3 or low-dose 5-fluorouracil 219 and cue a follow-up cryoablation, or to measure drug biodistribution after a cold-triggered release (**Figure 8**). Aside from MR, CT, or US imaging, non-conventional methods of imaging for cryoablation such as near infrared (NIR) fluorescence imaging with probe-conjugated nanoparticles or surface-enhanced Raman scattering with gold nanoparticles may be feasible to use with cryoablation. Optical imaging such as NIR and Raman imaging have some advantages over the non-optical imaging modalities, including better spatiotemporal resolution, sensitivity, and signal specificity. Nanoparticles loaded with image probe-conjugated drug can reveal tumor structures unhindered by the ice ball and characterize drug distribution in the tissues like the MRI-based theranostic nanoparticle discussed above. Combined with cryotherapy, these intraoperative imaging modalities using nanoparticles offer more accurate ablation of the tumor margins, lower chance of residual tumor, decreased rate of recurrence, and prolonged survival of patients.

### Multifunctional Carrier for Combination Cryoablation

Alongside the imaging properties, nanoparticles can carry therapeutic drugs for local delivery and minimize systemic toxicity. A spatiotemporal release of chemotherapeutic drugs complementing cryoablation can achieve a homogenous destruction of cancer cells and be used for tumors of larger sizes. Further, special care should be taken to allow drugs to be released from nanomaterials at the right time using thermosensitive polymers or degradable materials. Localized burst release of chemotherapy or immunotherapy on demand by applying cryoshock can concentrate drugs within tumor cells due to the formation of ice and increase cytotoxic effect 9. Burst release of chemotherapeutic drugs triggered by cold shock within cancer cells may overcome drug resistance by overloading the cells with drugs before efflux pumps of the cancer cells deplete them. Such strategies have been used to overcome drug resistance in ovarian cancer cells 221. A wide variety of polymers allows fabrication of nanoparticles with flexible and tunable properties. Genipin-cross-linked Pluronic F127-chitosan nanoparticles loaded with doxorubicin were both thermosensitive and pH-sensitive 222. Another type of polymeric nanoparticle, mPEG-poly(lactic-co-glycolic acid)-poly-L-lysine (mPEG-PLGA-PLL), can be loaded with doxorubicin and conjugated with cyclic RGD, a cell penetrating peptide that is also selective to tumor cells. mPEG-PLGA-PLL-cRGD nanoparticles have achieved selective targeting of tumor cells and improved survival of tumor-bearing mice 223. Wang et al. synthesized thermally responsive nanoparticles that triggered irinotecan (CPT) when subjected to cold temperatures 224. The nanoparticle synthesis was achieved through a double emulsion method (water in oil in water) to create chitosan modified F127 facing the hydrophilic outer and inner layer, with PNIPAM-B and irinotecan in the middle hydrophobic layer. Indocyanine (ICG) green was also added in the hydrophilic core for *in vivo* fluorescence imaging and to locally generate heat upon near-infrared radiation (NIR). When the temperature dropped below 10 °C, the PNIPAM-B polymer became hydrophilic, turning the water-in-oil-in-water (W/O/W) emulsion to water-in-water-in-water (W/W/W) emulsion. The nanoparticles disassembled to release irinotecan. They could also generate heat upon NIR for a photothermal effect. Importantly, *in vivo* fluorescence could be detected on IR images to confirm the co-localization of nanoparticles and ice formation.

Cryoablation itself triggers an immune response via tumor lysates and can be considered a weak form of *in situ* cancer vaccination. Therefore, there have been efforts to combine immunotherapy with cryoablation. Nanoparticle-based immunotherapy delivery systems have been explored for reducing the side effects of immunotherapeutic drugs as well as modulating the immunosuppressive tumor microenvironment for a robust activation of the immune system. Recent findings of “sticky” nanoparticles with many functional groups like carboxyl, hydroxyl and amine that capture tumor-associated antigens and DAMPs from ablated tumor cells show an efficient method of capture-and-delivery of the tumor lysates to antigen-presenting cells such as dendritic cells, which in turn travel to lymph nodes to activate T cells 225-227. Imaging, such as MRI, may be useful for validating that the nanoparticles have correctly migrated into the lymph node for the crosstalk between innate and adaptive immunity. These nanoparticles may be further supplemented with immune checkpoint inhibitors for tumor regression and powerful antitumor immune response. As most studies have been in more popular modalities of ablation such as microwave or radiofrequency ablation, there is a need to prove the concept in cryoablation. Since cryoablation is not heat-based, it is more likely to preserve protein structures of the antigens and result in a greater activation of adaptive immunity. **Figure 8** summarizes the translational applications of nanoparticles from imaging to therapy in cryoablation.

### Ideas for Multifunctional Nanomaterials in Cryoablation and Potential Therapeutic Strategies

**Figure 9:**
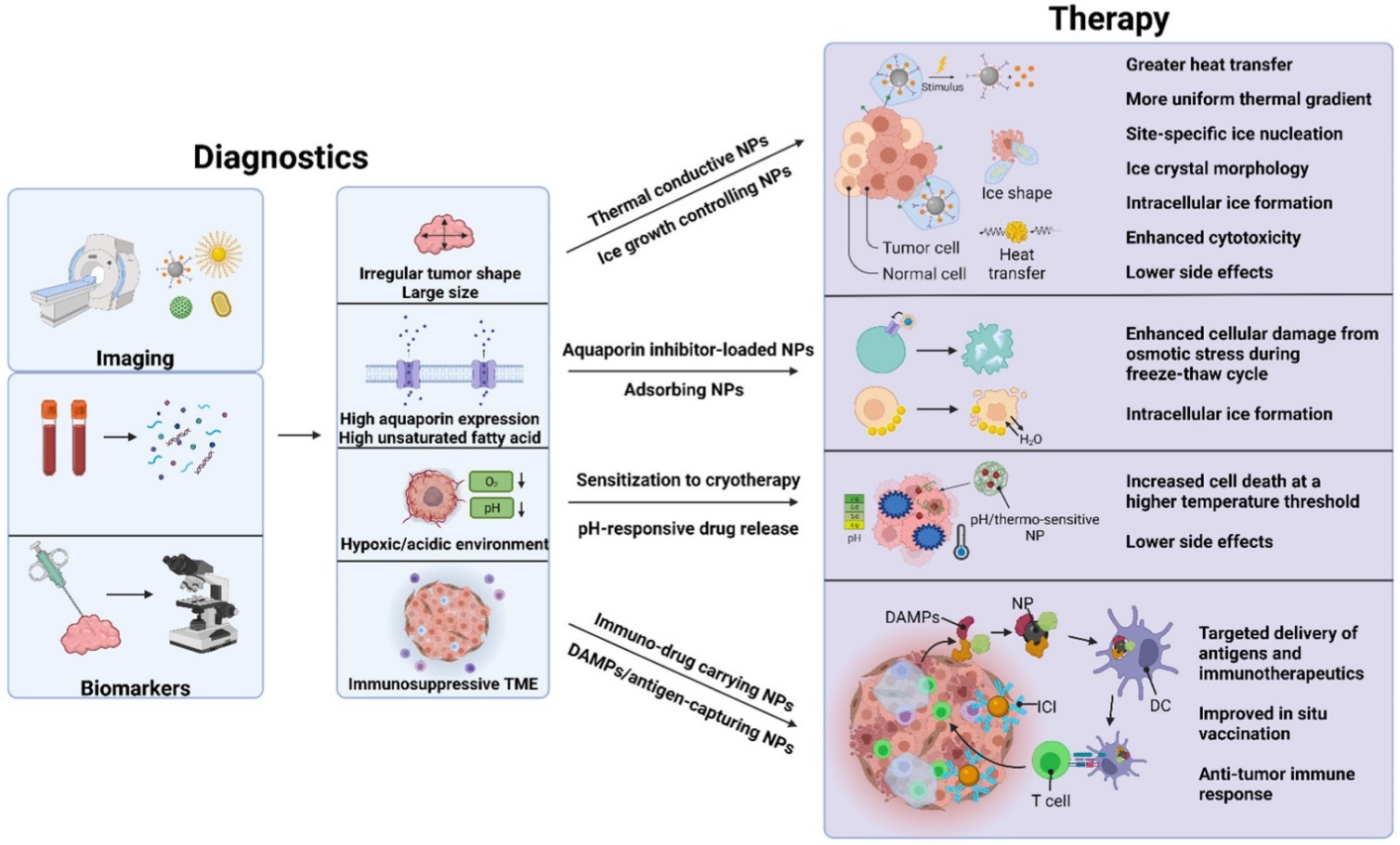
** Prospective therapeutic strategies for nanomaterials-based cryotherapy based on diagnosis of patients.** The central aim of cryoablation is to improve therapeutic efficacy while reducing side effects and tumor recurrence. Nanomaterials can help overcome current limitations of cryoablation itself and different tumors. Initial diagnosis can be done via imaging and/or biopsy to identify the tumor region and relevant information about the tumor including shape, size, membrane content, oxygen or pH levels, and immune profile of the microenvironment. After diagnosis, combination treatment can be performed with nanomaterials chosen to best target the limitations most efficiently. The design and therapeutic benefits of the nanomaterials can be considered as discussed in the paper.

Ideally, when designing multifunctional nanomaterials for use in a combination cryotherapy, fundamental parameters to look for are size, surface area, and surface functionalization. Nanoparticles with high surface area, as mentioned previously, can enhance heat transfer but also can increase more drug payload, which allows for lower number of nanoparticles and smaller drug dose. Functional groups decorated on the surface may also induce morphological change in ice crystals and affect affinity to the cell membranes. These factors should be based on what function of cryoablation or of nanomaterials to incorporate with cryoablation is desired. For instance, surface functionalization of a nanoparticle with imaging probes may confer high visualization of tumor but also affects drug loading efficiency or thermal conductivity, as well as nanoparticle-biomembrane interactions. Hence, it is important to consider what aspect of nanoparticles to utilize to enhance cryoablation. Their applications can be divided into diagnostic and therapeutic strategies. Diagnostic nanomaterials should be selected for their specificity and detection in cancer tissue. For types of cancers that are difficult to detect on traditional imaging platforms such as MRI, CT, or US, nanoparticles conjugated with contrast agents and molecular probes can be delivered into the tumor for precise ablation, as well as targeted therapy if adjuvants are additionally loaded onto the nanoparticles. Further, conditions of the cancers, such as size and shape of the tumor, and biomarkers for detecting expression of aquaporin and unsaturated fatty acids, low oxygen and pH levels, and immunosuppressive tumor microenvironment should be gathered from patients by imaging and liquid or tissue biopsy. Therapeutic nanoparticles can then be chosen based on the diagnostic result based on the discussions made in prior sections of this paper (**Figure 9**).

At the present, a theranostic platform combining both diagnostic and therapeutic potential of nanomaterials is an attractive venue for advancing cryoablation. In cryotherapy, uniformity and precision are the key to ensuring complete eradication of cancer cells while sparing healthy surrounding structures. Therefore, an ice nucleator that induces ice crystal formation at a specific site and imaging to confirm its location are needed. As mentioned in the previous sections, nanoparticles achieve these via modulation of its material properties. By targeting tumor biomarkers with nanoparticles, we can achieve high contrast imaging of the tumor structure which will be useful for ensuring that the ice formation covers the entirety of the tumor without overextension into healthy tissues. Concurrently, with localization of nanoparticles that possess properties to enhance ice nucleation and carry drugs, it becomes possible to achieve synergistic increase of cryotoxicity and therapeutic effect of the combination therapy while reducing the dosage of each drug. Hence, visual confirmation of nanoparticle deposition in the tumor via imaging and subsequent application of therapy are recommended. Ultimately, a horizontal integration of imaging and therapy is desirable, such that a theranostics nanomaterial can signal appropriate treatment after sensitizing the cancer cells to cryoablation, such as a nanoparticle that “turns on” once pH or O_2_ level is restored to normal to indicate for a subsequent radiotherapy. As of now, more investigations are necessary to systematically characterize the *in vivo* behaviors of theranostics nanoparticles to adjust the number of nanoparticles delivered to tumors sufficient for both diagnostic imaging accuracy and therapeutic effectiveness.

Next to identifying parameters for nanoparticle-mediated cryoablation, additional steps can be performed to make patients more suitable to nanomaterials. For instance, conditioning of tumor vasculature can increase permeability to nanoparticles or drugs. Gold nanoparticles conjugated with TNF-α increase intracellular ice formation and released TNF-α locally within the tumor regions for inflammatory response 228. The gold nanoparticles selectively accumulate in the tumor due to a ligand-receptor interaction between TNF- α and TNFR1 receptors overexpressed on tumor endothelial cells. Preconditioning of tumor vascular structures by TNF-α results in a disruption of endothelial barrier and hyperpermeability. Further, there are increased levels of fibrinogens which prolong vascular stasis. The combination immuno-cryotherapy has a synergistic effect, inhibiting tumor growth more than cryotherapy alone.

**Figure 10:**
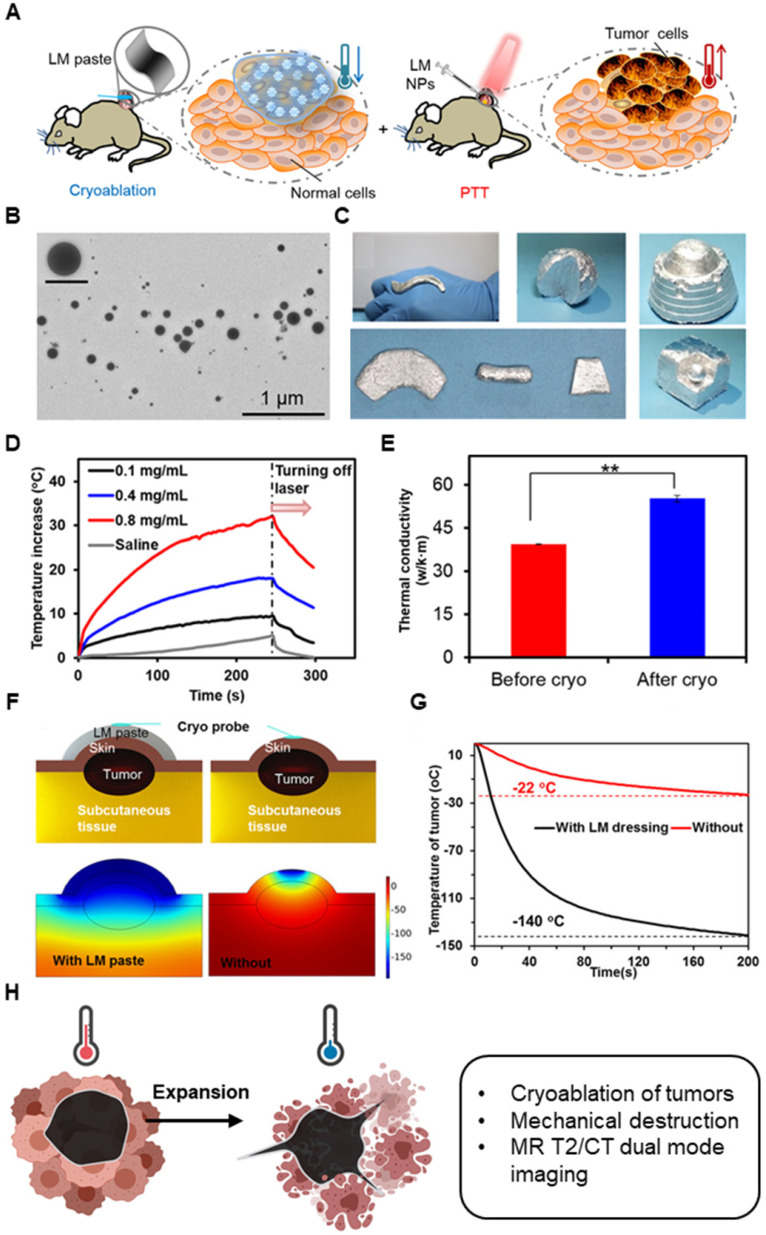
** Enhanced cryoablation using image-guided thermo-responsive gallium microparticles. (A)** Schematic of gallium liquid metal (LM) paste and LM nanoparticles used for cryoablation and photothermal therapy (PTT), respectively. **(B)** TEM images of LM nanoparticles. **(C)** Pictures of the LM paste showing its flexibility and formation into various shapes. **(D)** When irradiated with laser, the LM nanoparticle suspensions showed steeper temperature increases with increasing concentrations. **(E)** Thermal conductivity of the LM paste before and after freezing. **(F)** Graphical depictions of temperature gradient with or without the LM paste covering the tumor during cryoablation. **(G)** Simulated temperature of the tumor with or without the LM paste. Adapted and reprinted with permission from ref. 230. Copyright, 2020. American Chemical Society. **(H)** Schematic of gallium microparticles-based cryotherapy under CT and MR imaging. Gallium microparticles showed rapid extensions of sharp spikes upon freezing.

Finally, new types of materials and their intrinsic properties should be continuously explored for application in cryotherapy. A relatively new type of nanoparticle that has been garnering attention is liquid metal. Liquid metals such as gallium-based alloys have attracted considerable interest in the biomedical field due to their stability, high electrical and thermal conductivity, and non-toxicity 229. They are flexible and reformable at room temperature, allowing for easy manipulation in the lab. Taking advantage of high photothermal conversion efficiency and high thermal conductivity, Hou et al. used liquid metal nanoparticles-paste hybrid to combine cryoablation and photothermal therapy to treat melanoma (**Figure 10A-E**) 230. Gallium-indium alloy (GaIn) nanoparticles were injected subcutaneously in mice for photothermal therapy and the liquid metal paste (GaIn-Cu) was covered over the tumor to enhance the heat transfer during cryoablation. When applied with laser irradiation, GaIn nanoparticles displayed high heat transfer with increasing concentrations, while the liquid metal paste resulted in a rapid reduction of temperature and uniform heat distribution around the tumor (**Figure 10F-G**). The liquid metals-paste hybrid exhibited high thermal conductivity that increased during the cryoablation phase due to its phase transition from liquid to solid. Finally, although not strictly nanosized, gallium microparticles (~200 µm) exhibited sharp protrusions that pierced tumor cells upon freezing (**Figure 10H**) 231. Importantly, they showed clear visualization of tumor mass on both CT and T2 MR images as well as high thermal conductivity. Liquid metals that exhibit thermosensitive properties may be similarly applied to nanoparticles, and other materials that undergo a phase transition at cold temperatures may enable mechanical modulation of the tumor environment.

## Outlook and Conclusion

Minimally invasive tumor ablations are commonly used in the treatment of a broad range of tumors. Ablation therapies are one of many primary options for the patients who have failed chemotherapy or radiotherapy or are not surgical candidates. They have the potential to be first-line treatments for these patients at various stages of cancer. Thus, multiple ablative tools have been developed for a precise, minimally invasive removal of tumors or as an adjuvant tool to surgery. Radiofrequency (RF), microwave (MW), laser, and high-intensity focused ultrasound (HIFU) systems utilizing heat energy are well-established for the ablation of many types of tumors. However, tumor recurrence after the ablation and additional heat-related side effects continues to be an obstacle to better therapeutic outcomes. Compared to heat-based ablations, non-thermal ablation such as IRE and non-heat-based thermal ablation such as cryoablation have a better chance of minimizing damage to vascular structures and nearby organs. Further, cryoablation can be monitored real-time unlike all other ablation therapies, readily accomplishing high precision removal of tumor while preserving normal tissues. Moreover, it has accumulated years of clinical data and been deemed to be safe and easy to use. However, despite these benefits, cryoablation suffers from heterogenous tumor ablation, especially incomplete killing of tumor cells around the margins of the ice ball. Further, in cases where tumor is not readily visible by imaging, there is a risk of freezing healthy tissues. In addition, while cryoablation can induce anti-tumor immune response, this phenomenon by itself is insufficient for effective tumor regression and therefore must be supplemented with other forms of immunotherapy.

As immunotherapy becomes the main pillar of cancer therapy, the ablation therapies which can induce immunogenic cell death and antigen presenting cells or immune modulation will become paramount in achieving complete tumor response jointly with immunotherapy. Recent reports of cryoablation-mediated immune modulation strongly support the combination of cryoablation with various immunotherapies. Considering rapidly evolving essential imaging guidance and interventional oncology techniques, cryoablation and cryoablation-mediated combination therapy will be an exciting area in cancer therapy. For complete success of the treatment, image-guided approach can help clearly visualize ablation zone and assess complete tumor eradication. Real-time monitoring of both the ice and immunotherapeutic agents will be important in limiting systemic exposure, and post-ablation images can be assessed for signs of tumor progression or pseudoprogression occurring in less than 10% of the patients treated with immunotherapy 232. Course to treatment and recovery will likely entail utilizing immunotherapy and image-guidance together with cryoablation, rather than a monotherapy alone.

One promising platform to increase therapeutic efficacy and reduce side effects of cryoablation is multifunctional nanoparticles. Presently, nanoparticles are capable of loading of therapeutic chemicals and image contrasts due to their high surface area-to-volume ratio, while nanoparticles are image contrasts themselves. Nanocarriers have shown extensive potential as a catalyst of the ablation therapies, for supporting imaging guidance for minimally invasive procedure and precise ablation, and for further co-delivery of therapeutics that can allow various combination therapies, especially immunotherapies. As cryoablation is a local therapy, drug-eluting nanoparticles offer the benefit of localized chemo- or immunotherapy and can prevent systemic side effects, overcome drug resistance, and create synergy with cryotherapy. In addition, their high contrast imaging properties can provide accurate treatment and be used to track the biodistribution of drugs or nanoparticles. Recent studies demonstrated that nanoparticles could control different properties of ice ball formation, including rate of crystallization, probability of intracellular ice formation, and crystal morphology within tumor tissues for precise ablation targeting. Lipid-based nanoparticles, for example, can lower the freezing point and hamper ice growth while metallic nanoparticles can accelerate ice growth. Intravenously injected nanomaterials may additionally affect heat convection between vessels and the ablation zone. In summary, although there are currently very little clinical studies investigating nanoparticle-delivered therapeutics with cryoablation, cryoablation is a promising ablation technology that can achieve an innovative synergistic combination with other therapies. In conjunction with nanoparticles, image-guided combination cryotherapy has the possibility of eradicating cancer completely while preserving neighboring organs and tissues. Utilizing well-developed nanomedicine that supports hypothermic effects, image guidance and synergistic combinational immuno-therapeutics will play a critical role in extending cryoablation-based cancer therapies. Current clinical advances of cryoablation demonstrating precise ablation, strong immunogenicity and positive therapeutic outcomes are providing great potential to be one of the main ablation techniques that also can allow various combinational cancer therapies. Utilizing well-established nanomedicine and theranostic nanoparticles that can be used for cancer ablation techniques would be the best option to achieve an innovative therapeutic outcome by enhancing the tumor specificity and inducing anti-tumor immunogenicity. Further investigation of the nano-cryoablation therapies that have been relatively less studied is required for extending the therapeutic options of various types of tumors.

## Figures and Tables

**Table 1:** Commonly treated areas of cryoablation

Target Organs	Clinical phase	Number of articles	Benefits	Shortcomings (vs other methods)
Liver	Phase III	18	Larger and more precise zones of ablation 17; low complication rate for small tumors 18; minimal effect on surrounding organs 19	Correlation with inflammatory mechanisms in accessory organs 20-25; Higher risk of complications in large tumors 26
Prostate	Phase I/II	7	Preservation of sexual function 27; shorter recovery time 28	Local tumor recurrence after first cryoablation 29; effective as salvage, second-line treatment 30
Renal (T_1a_)	Phase II/III	9	Favorable OS/CSS for tumors ≤ 2cm 31-35; low rate of complications and superior renal functional preservation 36	Lower efficacy compared to partial nephrectomy for larger tumors (≥ 2 cm) 31
Renal (T_1b_)	Phase I/II	3	High progression-free survival (PFS) 37	Higher complication/mortality rate 38
Skin	Phase II/III	3	High cure rate; low probability of scarring	Skin burns, pigmentation changes
Breast	Phase II/III	5	Low rate of recurrence in small tumors (< 1.5cm); low probability of scarring	High complication rate in multifocal tumors; lower efficacy in larger tumors (> 2 cm)

Abbreviations: OS, overall survival; CSS, cancer-specific survival; PFS, progression-free survival.

**Table 2:** Cancer therapies combined with cryoablation and their clinical outcomes

	Types	Clinical Outcome Highlights
Chemotherapy	Zoledronic acid, sorafenib, 5-fluorouracil, gefitinib	Greater pain reduction with cryo-zoledronic acid vs monotherapy 75, 76; longer OS (RCC: 36 vs 29 months) for RCC 77 and HCC 78 when combined with sorafenib vs sorafenib only; greater tumor growth inhibition in cryo-5-fluorouracil vs monotherapy 79, 80; progression-free survival (PFS) and 1-year survival rate significantly greater than gefitinib alone 81
Immunotherapy	NK cells, pembrolizumab, CpG ODN 82, granulocyte macrophage colony-stimulating factor (GM-CSF) 83, 84, polysaccharide-K (Krestin), DC-CIK	Cryo-NK group had better PFS than cryo in HCC patients (9.1 vs 7.6 months) 85; enhanced T cell population near the tumor 86; combination of cryoablation and Krestin suppressed IL-4 and IL-10 production and marginally improved NK cell and cytotoxic T cell counts in splenocytes 87; Cryo-pembrolizumab resulted in high tumor mutational burden 88; higher median OS with Cryo-DC-CIK 89
Surgery	Pancreatic bypass, mitral valve surgery (maze), laparoscopy 90, resection 68	Improved median survival in pancreatic cancer patients with cryo-palliative bypass surgery vs. surgery alone (14 vs 8.5 months) 91; instances of post-operative bleeding were decreased in cryo-maze vs maze alone (6-8% to 3.4%) 92
Radiotherapy	Intensity-modulated radiotherapy (IMRT), conventional radiotherapy 93-96	No adverse events above Grade II in 5 patients with HCC treated by IMRT (5400 cGy/18f and 300 cGy/f) 97; significant pain reduction in cryo-radiotherapy group to treat bone metastases (20 Gy in 5 daily fractions following cryoablation) 98; Cryo with ^125^I a palliative treatment for cardiac metastasis 99 and results in higher survival times for pancreatic cancer patients 100, 101
Embolization	TACE 102, 103 (lobaplatin/epirubicin 104), TAE	Enhanced immune response from transcatheter renal arterial embolization 105; high technical success combining TAE and cryoablation via transradial access 106

**Abbreviations:** OS, overall survival; RCC, renal cell carcinoma; HCC, hepatocellular carcinoma; PFS, progression-free survival; NK cells, natural killer cells; CpG ODN, cytidyl guanosyl oligodeoxynucleotide; GM-CSF, granulocyte macrophage colony-stimulating factor; DC-CIK, dendritic cell-activated cytokine-induced killer cells; IL, interleukin; intensity-modulated radiotherapy, IMRT; cGy/f, centigrade in fractions; TACE, transcatheter arterial chemoembolization; TAE, transcatheter arterial embolization.

**Table 3:** Comparison of MRI, CT, and US

	Method	Specialty/Benefits	Drawbacks
MRI	MR fluoroscope for real-time; with or without a contrast agent	Correlated with favorable post-operative results; High soft tissue resolution; nonionizing; real-time monitoring	Expensive and long procedure; may require contrast agents for enhancement
CT	Conventional CT scanner or CT fluoroscope; with or without a contrast agent	Correlated with high technical success; soft tissue and skeletal visualization; real-time monitoring; operator-independent; deep tissue visualization	Ionizing radiation; may require contrast agents
US	Conventional ultrasound sonograph	Cheaper than MRI/CT; nonionizing; real-time monitoring	Operator dependent; image prone to degradation; limited tissue penetration

Abbreviations: MRI, magnetic resonance imaging; CT, computed tomography; US, ultrasound.

**Table 4:** Summary of nano/micromaterials used to enhance cryoablation, including type, thermal conductivity, therapeutic cargos, and compatible imaging type

Material type	Thermal conductivity	Therapeutic agents and other properties	Compatible imaging type	Ref
Fe_3_O_4_ NPs	High	Increased IIF	MRI	Ye et al. 188
Au NPs	High	TNF-α, increased IIF	CT	Shenoi et al. 228 Yan et al. 190
MgO NPs	High (34.3 W m^-1^ K^-1^)	Increased IIF		Di et al. 187
Ag NPs	High (417.5 W m^-1^ K^-1^)	Faster freezing	CT	Yan et al. 190
Al NPs	High	Increased ice ball growth		Yan et al. 192
CS-CNC	Low	Sharp, needle-like ice crystals, increased IIF, faster freezing		Hou et al. 198
F127-chitosan NPs	Low	Doxorubicin		Hou et al. 222
mPEG-PLGA-PLL-cRGD NPs	Low	Doxorubicin, targeting		Ye et al. 223
HA-chitosan-F127-PNIPAM-B NPs	Low	Irinotecan, indocyanine green	Fluorescence	Wang et al. 224
CS-TPP NPs	Low	Trehalose (cryoprotectant)		Yao et al. 194
Liquid metal NPs (GaIn-Cu composite)	High (~38 to ~58 W m^-1^ K^-1^)	Lower and uniform temperature distribution in the tumor	CT/MRI	Hou et al. 230
Ga MPs	High (13 W m^-1^ K^-1^)	Mechanical damage	CT/MRI	Sun et al. 231

Abbreviations: IIF, intracellular ice formation; Fe_3_O_4_ NPs, iron oxide nanoparticles; Au NPs, gold nanoparticles; MgO NPs, magnesium oxide nanoparticles; Ag NPs, silver nanoparticles; Al NPs, aluminum nanoparticles; CS-CNC, chitosan-decorated cellulose nanocrystal; mPEG-PLGA-PLL-cRGD NPs, methoxy poly(ethylene glycol)-poly(latic-co-glycolic acid)-poly-L-lysine-cylic RGD nanoparticles; HA, hyaluronic acid; PNIPAM-B, poly(N-isopropylacrylamide-co-butylacrylate); F127, Pluronic 127; CS-TPP NPs, chitosan-tripolyphosphate nanoparticles; GaIn-Cu, gallium-indium-copper.
